# Deregulated calcium signaling in blood cancer: Underlying mechanisms and therapeutic potential

**DOI:** 10.3389/fonc.2022.1010506

**Published:** 2022-10-18

**Authors:** Tracey Immanuel, Jixia Li, Taryn N. Green, Anna Bogdanova, Maggie L. Kalev-Zylinska

**Affiliations:** ^1^ Blood and Cancer Biology Laboratory, Department of Molecular Medicine and Pathology, University of Auckland, Auckland, New Zealand; ^2^ Department of Laboratory Medicine, School of Medicine, Foshan University, Foshan City, China; ^3^ Red Blood Cell Research Group, Institute of Veterinary Physiology, Vetsuisse Faculty, University of Zurich, Zürich, Switzerland; ^4^ Zurich Center for Integrative Human Physiology, University of Zurich, Zürich, Switzerland; ^5^ Haematology Laboratory, Department of Pathology and Laboratory Medicine, Auckland City Hospital, Auckland, New Zealand

**Keywords:** Calcium signaling, calcium homeostasis, blood cells, lymphoma, myeloproliferative neoplasms, red cell abnormalities, leukaemia, cancer biological pathways

## Abstract

Intracellular calcium signaling regulates diverse physiological and pathological processes. In solid tumors, changes to calcium channels and effectors *via* mutations or changes in expression affect all cancer hallmarks. Such changes often disrupt transport of calcium ions (Ca^2+^) in the endoplasmic reticulum (ER) or mitochondria, impacting apoptosis. Evidence rapidly accumulates that this is similar in blood cancer. Principles of intracellular Ca^2+^ signaling are outlined in the introduction. We describe different Ca^2+^-toolkit components and summarize the unique relationship between extracellular Ca^2+^ in the endosteal niche and hematopoietic stem cells. The foundational data on Ca^2+^ homeostasis in red blood cells is discussed, with the demonstration of changes in red blood cell disorders. This leads to the role of Ca^2+^ in neoplastic erythropoiesis. Then we expand onto the neoplastic impact of deregulated plasma membrane Ca^2+^ channels, ER Ca^2+^ channels, Ca^2+^ pumps and exchangers, as well as Ca^2+^ sensor and effector proteins across all types of hematologic neoplasms. This includes an overview of genetic variants in the Ca^2+^-toolkit encoding genes in lymphoid and myeloid cancers as recorded in publically available cancer databases. The data we compiled demonstrate that multiple Ca^2+^ homeostatic mechanisms and Ca^2+^ responsive pathways are altered in hematologic cancers. Some of these alterations may have genetic basis but this requires further investigation. Most changes in the Ca^2+^-toolkit do not appear to define/associate with specific disease entities but may influence disease grade, prognosis, treatment response, and certain complications. Further elucidation of the underlying mechanisms may lead to novel treatments, with the aim to tailor drugs to different patterns of deregulation. To our knowledge this is the first review of its type in the published literature. We hope that the evidence we compiled increases awareness of the calcium signaling deregulation in hematologic neoplasms and triggers more clinical studies to help advance this field.

## 1 Introduction

The deregulation of signaling by calcium ions (Ca^2+^) has been extensively studied in solid tumors (1, 2). Changes to Ca^2+^ channels and effectors *via* mutations or changes in expression affect many functional capabilities responsible for cancer growth, invasion, and metastasis (2–5). The function of the endoplasmic reticulum (ER), the main site of Ca^2+^ storage in a cell, and Ca^2+^ transfer from the ER to mitochondria, the main regulation point for apoptotic cell death, are often deregulated in solid tumors (6, 7). Our review presents the rapidly accumulating data that this deregulation appears similar in many types of blood cancer. Therapeutic opportunities targeting Ca^2+^ signaling are emerging for disorders such as leukemia, lymphoma, and myeloproliferative neoplasms (MPN) (8–11), but this information is not yet widely known. Therefore, to increase awareness, we provide an outline of core findings that demonstrate deregulation of Ca^2+^ signaling in blood cancer. Research in this field has accelerated enormously in recent years, therefore, despite our great efforts, this review is unlikely to be complete. Nevertheless, we hope our compilation of data makes the subject of abnormal Ca^2+^ signaling in blood cancer more widely known. To our knowledge, this is the first review of this type in the published literature.

### 1.1 Unique relationship between extracellular Ca^2+^ and hematopoietic stem cells

Ca^2+^ signaling regulates many cellular processes, including gene expression, cell proliferation, motility, apoptosis, enzyme activity, and cytoskeletal dynamics, all of which are crucial to supporting normal cell differentiation including of hematopoietic stem cells (HSCs) (12–14). Specific effects of Ca^2+^ signaling are achieved through a tight control of intracellular Ca^2+^ homeostasis. At the resting state, cytosolic Ca^2+^ concentrations are maintained at very low levels: ∼50–100 nM in most cells and reported to be as low as 20–30 nM in HSCs (15). This contrasts with high extracellular Ca^2+^ concentrations of ~1.5 mM in most fluids, including in blood plasma and bone marrow interstitial space (16). On the background of this high extracellular-intracellular Ca^2+^ gradient, precisely regulated spatio-temporal increases in cytosolic Ca^2+^ levels trigger signaling events (17).

The bone marrow environment provides a unique extracellular context for Ca^2+^ signaling. High Ca^2+^ levels in the endosteal niche have been shown to assist homing of HSCs through their calcium-sensing receptor (18). Nevertheless, it remains unclear if low or high Ca^2+^ concentrations are required to support HSC quiescence, both were shown to apply (14, 15, 19, 20). A recent study demonstrates that there is heterogeneity in Ca^2+^ levels between bone marrow cavities, depending on the level of bone resorption, but unexpectedly, no sharp gradient towards the endosteal niche was observed (16). HSCs reside in locations with higher extracellular Ca^2+^ levels compared to the serum and to the overall Ca^2+^ levels in the bone marrow. With aging, there is a significant increase in extracellular Ca^2+^ levels in the bone marrow associated with clonal expansion of activated HSCs. It has been proposed that deregulated Ca^2+^ homeostasis may be involved in leukemic transformation of HSCs, but experimental validation is required (21). In support, changes in Ca^2+^ homeostasis influence cancer stem cell properties in other cancer types (22).

### 1.2 Principles of intracellular calcium signaling

**Figure 1:**
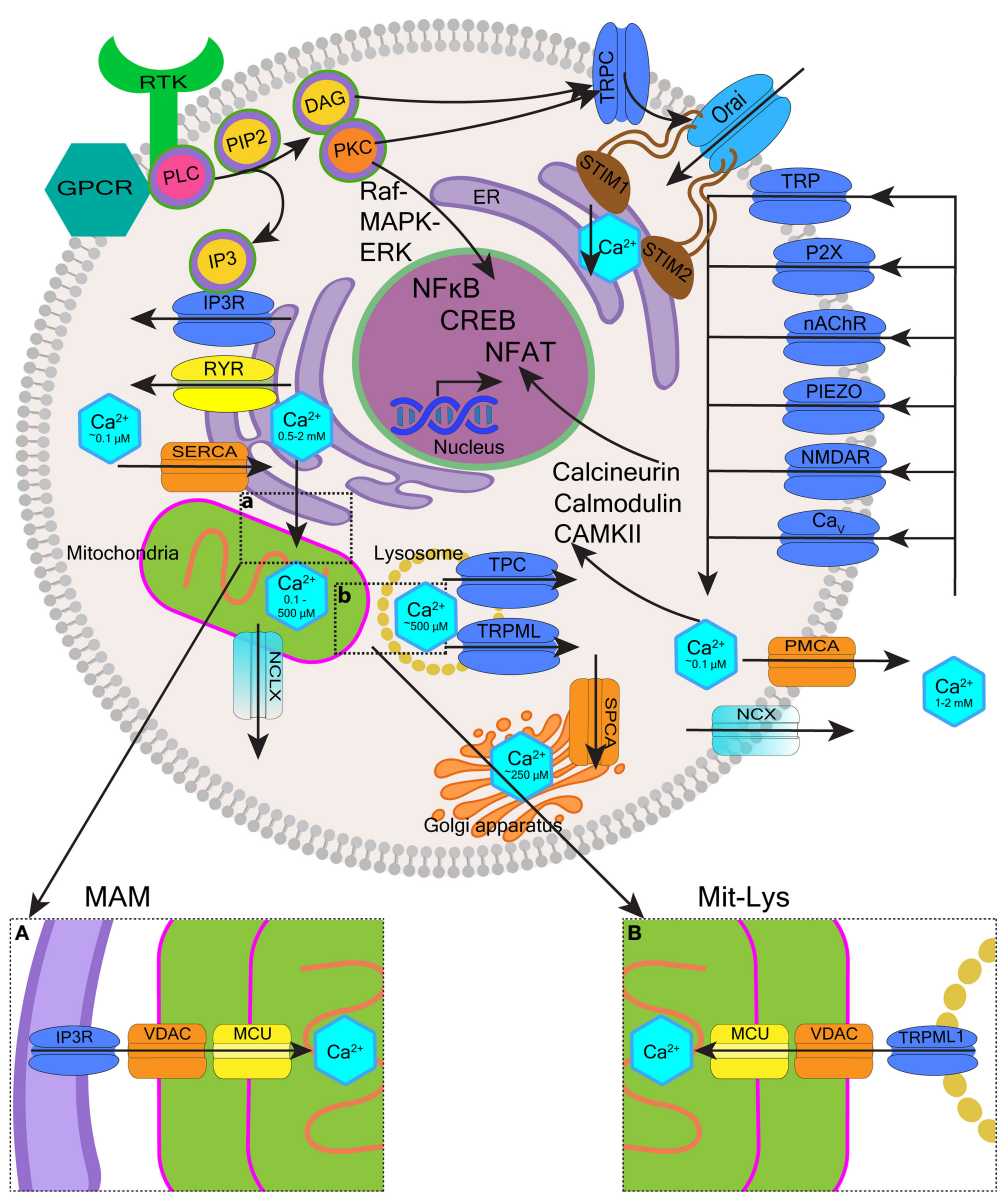
Overview of intracellular calcium homeostasis. Calcium homeostasis is maintained by the influx and efflux of Ca^2+^ through calcium channels and pumps located on the plasma membrane, as well as membranes of organelles such as the endoplasmic reticulum (ER), the Golgi apparatus, mitochondria, and endo-lysosomes. The cytoplasm, extracellular space, and each organelle have unique resting Ca^2+^ concentrations that have been indicated. Extracellular Ca^2+^ is transported into the cytosol through different channels such as transient receptor potential (TRP) channels, purinergic receptor (P2X) channels, nicotinic acetylcholine receptor (nAChR) channels, Piezo mechanosensitive channels, ionotropic glutamate receptor channels (e.g. N-methyl-D-aspartate receptors, NMDARs), and voltage-gated calcium (Ca_v_) channels. Ca^2+^ is removed from the cytosol to the extracellular space by plasma membrane calcium ATPase (PMCA) efflux pumps and sodium-calcium exchangers (NCX). Activation of cell surface transmembrane receptors with tyrosine-based activation motifs (RTK e.g. B-cell and T-cell receptors) or G-protein coupled receptors (GPCR e.g. neurokinin-1 receptor) activate phospholipase C (PLC). PLC hydrolyses phosphatidylinositol-4,5-bisphosphate (PIP2) located in the plasma membrane which generates two second messengers inositol 1,4,5-trisphosphate (IP3) and 1,2-diacylglycerol (DAG). IP3 binds to IP3 receptors (IP3Rs) located on the ER membrane leading to the release of Ca^2+^ from the ER and DAG activates protein kinase C (PKC). Depletion of ER Ca^2+^ activates stromal interaction molecules STIM1-STIM2 (located in the ER membrane), which then activates Orai1-Orai3 channels (located in the plasma membrane) to induce Ca^2+^ influx into the cytosol. This mechanism is called store-operated calcium entry (SOCE). Ryanodine receptors (RYRs) represent an alternative pathway for Ca^2+^ release from the ER regulated by Ca^2+^, Mg^2+^ and other molecules including ATP, calmodulin and CaMKII. The Ca^2+^ concentration in the ER is replenished *via* sarco-endoplasmic reticulum calcium ATPase 2b (SERCA2b) pump. The influx of Ca^2+^ from the ER to mitochondria occurs through voltage-dependent anion channels (VDAC) and mitochondrial calcium uniport (MCU) located in high numbers within mitochondria-associated ER membranes (MAMs) (**insert a**). Ca^2+^ leaves mitochondria mostly through Na^+^/Ca^2+^/Li^+^ exchanger (NCLX). Ca^2+^ stored in the endo-lysosomes is mobilized mostly by two-pore channels (TPC) and transient receptor potential mucolipin (TRPML) channels in response to nicotinic acid adenine dinucleotide phosphate (NAADP.) TRPML1 is involved in the mitochondrial-lysosomal contact sites (Mit-Lys), facilitating Ca^2+^ transfer to mitochondria through VDAC and MCU (**insert b**). Multiple effector molecules mediate effects of Ca^2+^ signaling including PKC, Raf-MAPK (mitogen-activated protein kinase)-ERK (extracellular signal-regulated kinase), calmodulin, calcium/calmodulin-dependent protein kinases (e.g. CaMKII), and calcineurin. These signaling molecules influence gene expression through transcription factors such as nuclear factor kappa B (NF-κB), cyclic adenosine monophosphate (cAMP) response element-binding protein (CREB), and nuclear factor of activated T-cells (NFAT).

Cytoplasmic free Ca^2+^ levels are maintained by Ca^2+^ buffer systems (23) and modulated by a system of molecules re-distributing Ca^2+^ between the intracellular stores (the ER, mitochondria, Golgi apparatus and lysosomes), taking Ca^2+^ in from the extracellular space, or extruding it from the cell (12). Various channels, exchangers and pumps regulate Ca^2+^ levels in cells, including in blood cells. The collective involvement of these molecules, often referred to as a Ca^2+^-signaling toolkit (13, 24), is shown in **Figure 1** **(**with molecular details described in the figure legend).

In this review, we wish to highlight the role of ER as the main site of Ca^2+^ storage in almost any cell, as this functionality is often deregulated in cancer including blood cancer (25). The concentration of free Ca^2+^ in the ER is ~500 μM and ~2 mM for total ER Ca^2+^, most of which is bound to Ca^2+^-binding proteins such as calreticulin (CALR) (26). Many pathways of cell activation converge on the efflux of Ca^2+^ from the ER that occurs through channels called inositol 1,4,5-trisphosphate (IP3) receptors (IP3Rs) (27) (**Figure 1**). IP3Rs induce the release of Ca^2+^ from the ER upon binding of IP3 generated by phospholipase C (PLC) (28). PLC operates downstream of G-protein coupled receptors (GPCRs) and tyrosine kinase receptors located in the plasma membrane (29, 30). When ER Ca^2+^ becomes depleted, extracellular Ca^2+^ influx is initiated to maintain signaling. In non-neuronal cells most extracellular Ca^2+^ enters the cell through the mechanism called store-operated calcium entry (SOCE) (31, 32). SOCE is triggered by stromal interaction molecules (STIM1-STIM2) located in the ER membrane. Upon sensing ER Ca^2+^ depletion, STIM proteins oligomerize and redistribute to the plasma membrane where they interact with Orai1-Orai3 channels to activate Ca^2+^ influx into the cytosol (26, 32) STIM2 has low affinity for Ca^2+^ and activates when ER Ca^2+^ stores are <500 μM. In contrast, STIM1 has high affinity for Ca^2+^ and only activates when Ca^2+^ stores are <300 μM (33). Loss of STIM2 occuring in certain cancers is thought to reduce ER Ca^2+^ content (7). PLC also generates 1,2-diacylglycerol (DAG) that performs its signaling functions by binding and activating other proteins, including protein kinases C (PKC) and certain transient receptor potential (TRP) canonical (TRPC) channels, in particular TRPC1, that can interact with Orai1 and STIM1 to support SOCE (29, 34) (**Figure 1**).

Ca^2+^ transfer from the ER to mitochondria is another important mechanism often hijacked in solid tumors and of emerging importance in blood cancer (21, 35). ER and mitochondria interact through specialized ER-mitochondrial contact sites called mitochondria-associated ER membranes (MAMs) (36) (**Figure 1**, insert a). Within MAMs, IP3Rs on the ER interact with voltage-dependent anion channels (VDACs) located in the outer mitochondrial membrane allowing unrestricted Ca^2+^ entry into the inter-membrane space (37). The passage of Ca^2+^ through the inner mitochondrial membrane is restricted by the mitochondrial calcium uniport (MCU) and the membrane potential (ΔΨm ∼ −150 mV) (38). Small amounts of mitochondrial Ca^2+^ support mitochondrial metabolism, providing a mechanism that couples cellular activity with the generation of adenosine triphosphate (ATP). Ca^2+^ uptake into mitochondria activates pyruvate dehydrogenase, α-ketoglutarate dehydrogenase, and isocitrate dehydrogenase, thereby stimulating the tricyclic acid cycle and energy generation (39, 40). In contrast, high levels of Ca^2+^ in the mitochondria induce apoptosis (41, 42). Prolonged accumulation of Ca^2+^ in the mitochondria leads to the opening of the mitochondrial permeability transition pore (mPTP) formed when VDAC1 clusters with adenine nucleotide translocase (on the inner mitochondrial membrane) and cyclophilin D (in the mitochondrial matrix). The mPTP opening causes depolarization of the inner mitochondrial membrane, which uncouples the respiratory chain leading to increased mitochondrial membrane permeability and the release of cytochrome c (21, 35, 36).

Oncogenic effects have also been shown for certain endo-lysosomal Ca^2+^ storage and release mechanisms (43). Endo-lysosomes are heterogenous and dynamic acidic organelles that in addition to other roles, act as intracellular Ca^2+^ stores (44, 45). Endo-lysosomes sequester and release Ca^2+^ to the cytosol mainly through two-pore channels (TPC1-TPC2) and TRP mucolipin channels (TRPML1-TRPML3) activated by second messengers such as nicotinic acid adenine dinucleotide phosphate (NAADP), the most potent Ca^2+^-mobilizing second messenger known (46, 47). Effects of endo-lysosomal Ca^2+^ release may be both local and global. The latter occur when endo-lysosomal mechanisms act in conjuction with the ER to induce or inhibit ER Ca^2+^ release (48). Endo-lysosomal Ca^2+^ signaling regulates processes such as membrane trafficking, vesicle fusion and secretion which impacts a range of cellular behavious e.g. immune responses, autophagy, cell proliferation, and migration (43, 49). In analogy to MAMs, mitochondrial membrane contact sites have also been shown to involve lysosomes (50, 51) (**Figure 1**, insert b). The release of lysosomal Ca^2+^ through TRPML1 supports Ca^2+^ transfer to mitochondria, providing an additional mechanism through which intracellular Ca^2+^ signaling, mitochondrial bioenergetics and lysosomal effects can be regulated (51).

This review emphasizes importance of abnormal Ca^2+^ signaling in hematologic cancers. We begin by presenting the long-standing foundational data on Ca^2+^ homeostasis in red blood cells (RBCs) as historically, this work provided guidance for research into Ca^2+^ signaling in selected blood cancers. We then focus on the neoplastic impact of deregulated Ca^2+^ influx through the plasma membrane and the ER, Ca^2+^ efflux *via* Ca^2+^ pumps and exchangers, and the impact of deregulated Ca^2+^ sensor and effector proteins in blood cancer. Throughout the review we highlight potential therapeutic strategies being developed to abrogate this deregulation.

## 2 Foundational research into calcium signaling in red cells with an outline of the toolkit components

**Figure 2:**
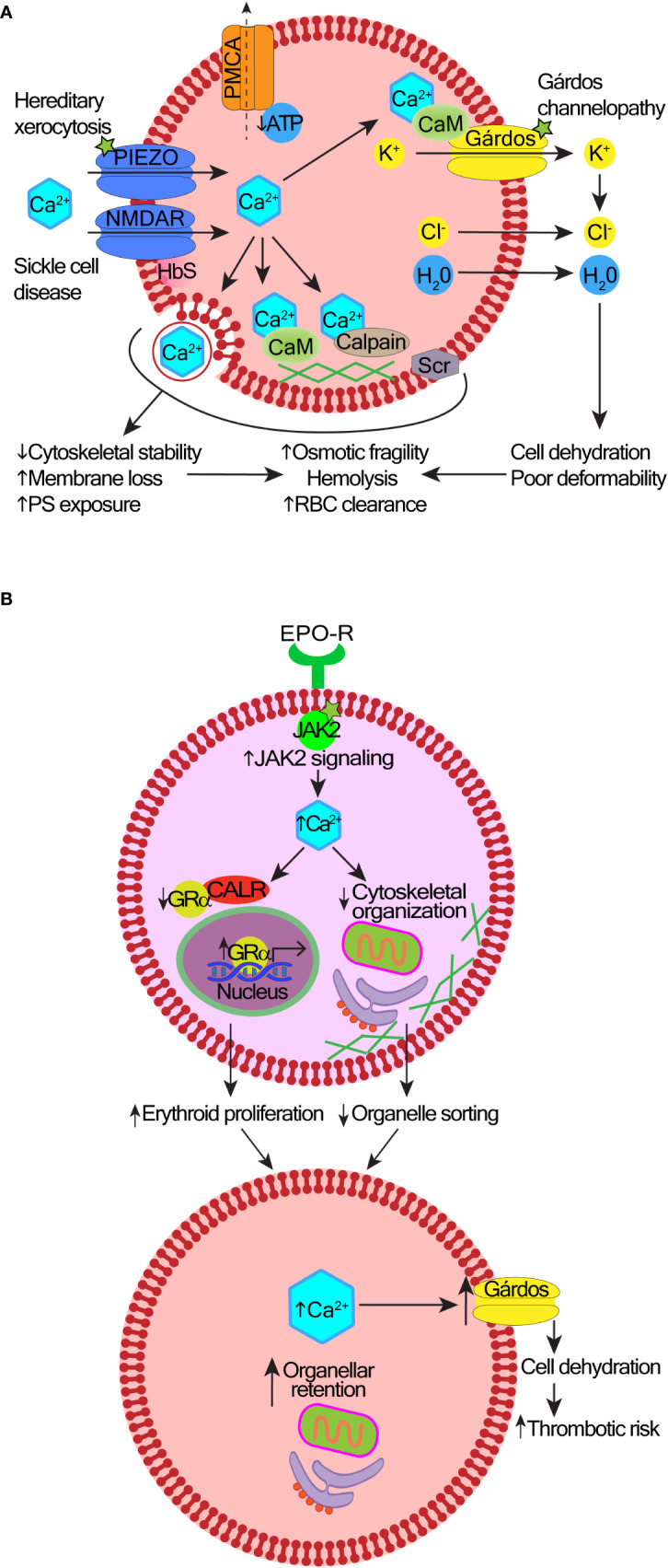
Mechanisms and consequences of deregulated calcium signaling in red cells and erythroid precursors. **(A)** In hereditary stomatocytosis/xerocytosis, heterogenous gain-of-function mutations in the Piezo1 or Gárdos channels cause excessive Ca^2+^ entry into red blood cells (RBCs). In sickle cell disease, there is an abnormally high abundance and activity of NMDA receptor channels, and probably other Ca^2+^-transporting ion channels contributing to the increased permeability of the RBC membrane to Ca^2+^. A layer of aggregated hemoglobin S (HbS) interferes with the shedding of NMDA receptor channels from the cell surface. Reduced levels of adenosine triphosphate (ATP) impair the function of the plasma membrane calcium pump (PMCA); as a result, Ca^2+^ uptake exceeds its efflux. Excess intracellular Ca^2+^ can be sequestered into vesicles and extruded, protecting the cytosolic and membrane proteins from Ca^2+^-induced damage. However, over time, increased cytosolic Ca^2+^ overactivates the Gárdos channel leading to cell dehydration. Membrane and cytoskeletal instability are induced by the overactive calcium/calmodulin (CaM) complexes, calpain, or scramblase (Scr). This leads to premature RBC clearance, hemolysis, and anemia. The exact contribution of these mechanisms to different types of anemia remains under investigation. **(B)** Effects of high intracellular Ca^2+^ in polycythemia vera (PV). Hyperactive JAK2 V617F mutation increases Ca^2+^ levels in erythroid precursors. Ca^2+^ overload impairs the nuclear export function of calreticulin (CALR), which results in nuclear retention of the glucocorticoid receptor α (GRα) responsible for stress response and erythroid proliferation. Defective organelle sorting and extrusion from erythroblasts leaves organellar remnants in reticulocytes.

Research into Ca^2+^ homeostasis in RBCs has a long history and has been regularly reviewed (52–54). While reticulocytes and immature RBCs of patients with sickle cell disease retain some of the mitochondria (55), normal mammalian RBCs do not have true Ca^2+^ storage organelles. However, RBCs often contain inside-out vesicles that are formed in response to increased Ca^2+^ uptake. These vesicles contain plasma membrane calcium ATPases (PMCAs) that pump Ca^2+^ from the cytosol into vesicles and thus protect the cytosolic and membrane proteins from Ca^2+^-induced damage (oxidation, proteolysis, irreversible dehydration) (see **Figure 2A** and the corresponding legend for molecular details). Some of these Ca^2+^-filled vesicles are extruded, other inside-out endosomes are retained inside the cells (56–58). The resting concentration of cytosolic Ca^2+^ in RBCs are similar to nucleated cells, ranging from 30–60 nM in normal RBCs to the pathological 300 nM levels in patients with certain hereditary anemias (59). This compares with 1.2-1.8 mM in blood plasma (52, 53). Cytosolic Ca^2+^ concentrations in RBCs affect many aspects of red cell physiology including cell hydration, metabolic activity, redox state, and proteolysis. Regulation of Ca^2+^ concentrations translates into the control over the remodeling of the cytoskeletal elements and concomitant changes in cell shape, cell volume, rheological properties and ultimately, RBC longevity and clearance (52) **(** **Figure 2A**).

Multiple types of channels permeable for Ca^2+^ are present in the RBC membrane supporting versatility and plasticity of intracellular Ca^2+^ signaling (53) (**Figure 2A**). These channels are present in RBCs in very low copy numbers to keep the basal Ca^2+^ permeability of the plasma membrane low. Each channel type responds to its own stimulus (e.g. mechanical, electrical or chemical) to induce Ca^2+^ oscillations under specific conditions. Due to the broad variance in channel copy number per cell, there is variation in RBC responses to stimulation, and the numbers of “responding cells” typically range from 10% to 30% (60–62).

One of the first Ca^2+^ signaling processes identified in RBCs was the function of the Gárdos channel (potassium calcium-activated channel subfamily N member 4, KCNN4) (63) (**Figure 2A**). KCNN4 is activated by Ca^2+^ that enters through any of the non-selective cation channels [e.g. piezo type mechanosensitive ion channel component 1, Piezo1 (64)]. Piezo channels are the largest plasma membrane Ca^2+^ channels known containing a three-bladed propeller-shaped structure that spans the lipid bilayer sensing membrane stretch (65–67). The activation of Piezo links mechanical forces applied to RBCs with the control of cell volume and lifespan (64, 68). The KCNN4 activation leads to K^+^ efflux and water loss (69), which reduces RBC volume and facilitates cell shape change. Activation of KCNN4 in RBCs of healthy people most likely enables better passage of RBCs through narrow capillaries (70), while its overactivation causes Ca^2+^-overload and RBC dehydration (71) **(** **Figure 2A** **)**. Hereditary stomatocytosis/xerocytosis are caused by gain of function mutations in genes encoding either KCNN4 (58, 72, 73) or Piezo1 channels (74–76). Different mutations cause distinctive clinical phenotypes, including some with syndromic features (72). The increasing use of next-generation-sequencing will help characterize the scope of genetic variants that are clinically relevant.

Other Ca^2+^ channels in RBCs include selected TRP and voltage-gated Ca^2+^ channels (Ca_v_), *N*-methyl-D-aspartate (NMDA) receptors, and VDACs (52, 53). The TRP channels are a large family of approximately 30 structurally related but diverse members, the majority of which function as non-selective cation channels with variable Ca^2+^ permeability (77, 78). TRP channels can be activated by multiple external ligands including inflammatory and pain mediators, certain spices (e.g. garlic, mint, camphor and chili), metabolites, or physical stimuli such as temperature and stretch. TRP channels act as environmental sensors and transduction channels that regulate intracellular Ca^2+^ levels in response to the depletion of internal Ca^2+^ stores with or without simultaneous activation by PLC (79–81). The importance of TRP and Piezo channels in human physiology and pathology is underscored by the award of the Nobel Prize in Physiology or Medicine in 2021 to David Julius and Ardem Patapoutian “for their discoveries of receptors for temperature and touch” (82–84). Based on the amino acid sequence homology, activation mode and function, TRP channels are divided into six subfamilies: TRPC (canonical, TRPC1-TRPC7), TRPV (vanilloid, TRPV1-TRPV6), TRPM (melastatin, TRPM1-TRPM8), TRPA (ankyrin, TRPA1), TRPML (mucolipin, TRPML1-TRPML3), and TRPP (polycystin, TRPP1-TRPP2) (77). All TRP channel types are tetrameric assemblies of subunits containing six transmembrane domains arranged around a central ion permeation pore (79).

All TRP channels mediate receptor-operated Ca^2+^ entry but some also function as components or regulators of SOCE (85, 86). The latter applies mostly to TRPC1 and TRPC4 as they can interact with and be activated by STIM1 upon depletion of the ER Ca^2+^ stores; in turn, TRPC1, TRPC3 and TRPC6 can interact and activate Orai1 channels to support ER Ca^2+^ store refilling (87). Other TRPC channels do not interact with STIM1 directly, however heteromeric assemblies combining TRPC1 with TRPC4/5 or TRPC3 with TRPC6/7 contribute to SOCE, implying single TRPC components can provide SOCE regulation (88). Other TRP channel types (e.g. TRPV4 and TRPV6) and other proteins also interact with TRPC channels, which influences diversity of their functioning (89).

TRPC6 is abundant in human RBCs and contributes to stress-stimulated Ca^2+^ entry but its specific function in RBCs remains elusive (90). The discovery of TRPV2 in RBCs is relatively recent (91). Similar to Piezo1, TRPV2 mediates Ca^2+^ influx into RBCs in response to mechanical activation, which modulates RBC osmotic fragility and may contribute to the RBC storage lesion (92).

NMDA receptors are ligand-gated non-specific cation channels with high Ca^2+^ permeability activated by glutamate and glycine (93). NMDA receptors play critical functions in the brain but are also expressed in non-neuronal cells, including all types of blood cells: red cells (60, 94, 95), platelets (96–98), neutrophils (99), monocytes (60), and lymphocytes (100, 101). In RBCs, NMDA receptor regulates hemoglobin oxygen affinity, nitric oxide production, cell hydration status, and proliferation of erythroid precursors (95). RBCs from patients with sickle cell disease carry higher numbers of NMDA receptors than in healthy donors (**Figure 2A** **)**. NMDA receptor overactivity leads to Ca^2+^ overload, K^+^ loss, cell dehydration, and oxidative stress, which may contribute to sickle cell crises (94). The efficacy of NMDA receptor inhibitor memantine for symptomatic treatment of sickle cell disease is currently being explored (102, 103).

VDACs are the major components of the outer mitochondrial membrane but they are also present in the plasma membrane including in RBCs (104, 105). VDACs conductance and selectivity are voltage-dependent. In the plasma membrane, VDACs may be involved in the transmembrane electron transport (37, 104). VDACs permeability for Ca^2+^ is low but considering the large intracellular-extracellular Ca^2+^ gradient, their activation may still contribute significant Ca^2+^ influx (53).

Finally, voltage-gated Ca^2+^ (Ca_V_) channels transduce changes in the plasma membrane potential to intracellular Ca^2+^ transients that initiate many crucial physiological processes (106). In neurons and muscle cells Ca_V_ channels primarily regulate synaptic transmission and contraction respectively but these channels also regulate secretion and biochemical processes such as enzyme activity, protein phosphorylation/dephosphorylation, and gene expression in other cell types. Ca_V_ channels are subdivided into Ca_V_1, Ca_V_2, and Ca_V_3 (107). Ca_V_2.1 is epressed in RBCs but its function is poorly defined (108).

Overall, Ca^2+^ channels play important roles in RBC membrane transport, metabolism, volume, shape and lifespan regulation, although many specific functions remain unknown (53). It has been proposed that increased Ca^2+^ levels in RBCs due to abnormal function of Ca^2+^ channels represents a common mechanism underlying an accelerated clearance of RBCs from the bloodstream and pathological hemolysis in a range of anemias, which is a new area for investigation (59).

## 3 Calcium signaling in normal and neoplastic erythropoiesis

The role of intracellular Ca^2+^ signaling during erythropoiesis has been recently reviewed (54). Ca^2+^ signaling regulates erythroid progenitor proliferation, differentiation, survival, and terminal enucleation. Changes in Ca^2+^ homeostasis are seen in reactive ineffective erythropoiesis (e.g. in β-thalassemia) (109) or in neoplastic erythropoiesis driven by Janus kinase 2 (JAK2) V617F mutation in polycythemia vera (PV) (110, 111). CALR is an ER-resident protein that regulates functions of other proteins by chaperoning them to their active sites in response to changing intracellular Ca^2+^ levels (112). In normal erythroid precursors, CALR promotes the nuclear export of glucocorticoid receptor α, which resets precursor proliferation to differentiation (110). In contrast, hyperactive JAK2 signaling in PV increases free intracellular Ca^2+^ levels, which impairs the nuclear export function of CALR (**Figure 2B**). Glucocorticoid receptor α is retained in the nucleus maintaining the expression of stress genes that increase proliferation of erythroblasts (110). Elevated levels of Ca^2+^ may also impair actin reorganization required to extrude organelles during enucleation (111). Consequently, PV reticulocytes have a high content of organellar remnants e.g. mitochondria, ER and ER-associated proteins including CALR. In mature RBCs from PV patients, high Ca^2+^ levels increase the activity of the Gárdos channel leading to cell dehydration (111) (**Figure 2B**).

Increased levels of cytoplasmic Ca^2+^, cell dehydration and the presence of organelle remnants in RBCs have the potential to promote thrombosis in PV (111). Dehydrated RBCs are more rigid, thus less amenable to shape changes required to pass through narrow capillaries, and also more susceptible to hemolysis under high-shear rates that occur in arterial circulation (113, 114). Higher cytoplasmic Ca^2+^ levels are known to increase adhesion between RBCs (115), and of RBCs to the endothelium (116, 117). Most previous work into PV-associated thrombosis focused on the role of a high hematocrit, white cell and platelet activation, coagulation factors and inflammation (118). However, a recent study used a laser-assisted optical rotational red cell analyzer to demonstrate abnormal RBC morphodynamics in 48 patients with PV (119). The deformability and stability of RBCs were reduced and RBC aggregation was increased. These alterations correlated with the incidence of ischemic stroke in 13 of these patients, suggesting a link between abnormal RBC morphodynamics and the increased risk of arterial thrombosis in PV, although this requires confirmation in larger studies (119).

Collectively, emerging data highlight a possible connection between the JAK2 V617F mutation and deregulated Ca^2+^ signaling in PV RBCs and precursors, with the potential to contribute to autonomous erythropoiesis and thrombosis. Therefore, strategies to modulate Ca^2+^ signaling may be useful for PV treatment.

## 4 Calcium signaling deregulation in blood cancer

Similar to solid tumors (3, 120), many blood cancers remodel Ca^2+^ signaling to promote their cancerous properties. Altered expression or activity of Ca^2+^ channels, pumps, and effectors can lead to the activation of transcription factors involved in the control of cell survival and proliferation.

### 4.1 Plasma membrane calcium-permeable channels

A number of Ca^2+^ influx channels located on the plasma membrane have been reported to impact on leukemic cells. These include the non-selective cation channels such as the TRP family, purinoreceptors (P2X7), nicotinic acetylcholine receptor (nAChR), Piezo1, NMDA receptor, and the Ca^2+^ selective Orai1 channels (**Figure 1**). **Table 1** provides a summary of such changes in different blood cancers, and an explanation of their functional effects follows.

**Table 1:** The differential expression of plasma membrane calcium channels and their relative contribution to the malignant phenotype in different blood cancers.

Cancer type	Molecule	Change in disease	Functional effects	References
AML	P2X7	↑ expression	↑ Ca^2+^ influx ^(C,P,M)^, ↑ proliferation ^(C,M)^, ↓ proliferation ^(C,P)^, ↓ remission rate ^(P)^, ↓ overall survival ^(P,M)^, altered sensitivity to chemotherapy ^(P,M)^, ↑ migration ^(M)^	(121–126)
	TRPV2	↑ expression	↑ proliferation ^(C)^, ↓ apoptosis ^(C)^	(127)
		↓ expression ^(P)^		(128)
	TRPM2	↑ expression	↑ proliferation ^(C)^, ↑ autophagy ^(C)^, ↑ mitochondrial Ca^2+^ influx ^(C)^, ↓ ROS production ^(C)^	(129, 130)
	TRPM4	↑ expression	↑ proliferation ^(C)^, cell cycle progression ^(C)^	(131)
	Orai1	↑ expression	↑ proliferation ^(C)^, ↑ migration ^(C)^, ↑ cell cycle progression ^(C)^	(132–134)
	IP3R2	↑ expression	↓ overall survival ^(P)^, ↓ event-free survival ^(P)^	(135)
	Ca_V_1.1 (CACNA1S) Ca_V_1.2 (CACNA1C)	↑ expression ^(P)^		(136, 137)
	Ca_V_1.2 (CACNA1C)	↓ expression in AML-MSCs	↑ AML proliferation in 2D and 3D co-culture models ^(P)^	(138)
ALL	P2X7	↑ expression	↑ Ca^2+^ influx ^(C)^,↑relapse ^(P)^	(121, 123)
		↓ expression ^(P)^		(124)
	TRPC4/C5	↑ expression ^(C)^		(139)
	TRPV5/V6	↑ expression	cell cycle progression ^(C)^, endocytosis ^(C)^, cell migration ^(C)^	(140–142)
	TRPM2	↑ expression	↑ Ca^2+^ influx ^(C)^, ↓ ROS production ^(C)^	(130, 143)
	TRPM4		abnormal Ca^2+^ oscillation pattern ^(C)^, ↓ cytokine secretion ^(C)^	(144)
	Orai1	↑ expression ^(P)^		(134)
	NMDAR	mutations in *GRIN2C*	↑ relapse in high-risk pediatric patients ^(P)^; effects on expression and functional consequences are unknown	(145)
	Ca_V_1.1 (CACNA1S) Ca_V_1.2 (CACNA1C)	↑ expression ^(P)^		(136, 137)
CML	P2X7	↑ expression	↓ remission rate ^(P)^	(121)
		↓ expression ^(C)^		(125)
	TRPV2	↑ expression	↑ proliferation ^(C)^, ↓ apoptosis ^(C)^	(127, 146, 147)
	TRPM2	↑ expression ^(C)^		(130)
	TRPM7	↑ expression	↑ Ca^2+^ influx ^(C)^, ↑ proliferation ^(C)^, differentiation ^(C)^	(146)
	Piezo1	↑ expression ^(C)^		(148)
	Orai1	↑ expression ^(C)^		(134)
CLL	P2X7	↑ expression	↑ Ca^2+^ influx ^(P)^, ↓ proliferation ^(P)^	(149)
	TRPC1	↑ expression	↑ cytokine secretion ^(P)^	(150)
	α7-nAChR	↑ expression	↑ proliferation ^(C)^, ↑ migration ^(P,C)^	(151)
	Orai1	↑ expression	↑ Ca^2+^ influx ^(P)^, ↓ event-free survival ^(P)^, ↓ progression-free survival ^(P)^	(11, 134)
	CACNA1A	↑ expression ^(P)^		(136, 137)
DLBCL	TRPM4	↑ expression	↓ overall survival ^(P)^, ↓ progression-free survival ^(P)^	(152)
	IP3R2	↑ expression	↑ sensitivity to BIRD-2-mediated cell killing ^(C)^	(153–155)
	Ca_V_1.1 (CACNA1S) Ca_V_1.2 (CACNA1C) Ca_V_1.3 (CACNA1D) Ca_V_1.4 (CACNA1F)	↑ expression of CACNA1D in ABC-DLBCL and of CACNA 1S, 1D and 1F in GCB-DLBCL ^(P)^		(156)
	Orai1	↑ expression ^(C,P)^		(134, 156)
Plasma cell myeloma	TRPV1	↑ expression	↑ proliferation ^(C,P)^, ↑ cell survival ^(C,P)^, ↑ drug resistance ^(C)^	(157, 158)
	TRPV2	↑ expression	↓ overall survival ^(P)^, ↓ event-free survival ^(P)^, ↑ bone lesions ^(P)^, ↑ cytokine secretion ^(P)^	(159, 160)
	TRPM7	↑ expression	↑ migration ^(C)^	(161)
	TRPM8	↑ expression ^(P)^		(162)
	TRPML2	↑ expression	↑ sensitivity to ibrutinib and/or bortezomib ^(C)^	(163)
	α7-nAChR	↑ expression	↑ proliferation ^(C)^, ↑ migration ^(C)^	(151)
	Orai1	↑ expression	↑ migration ^(C)^, ↓ progression-free survival ^(P)^	(161, 164)
Waldenström macroglobulinaemia	TRPC1	↓ expression ^(P)^		(165)

(P) = Patient cells, (C) = Cell lines, (M) = Mouse model. ↑ = increased, ↓ = decreased. Expression changes are often found in particular cell lines or leukemic subtypes and not in others. Empty cells indicate there is no data. AML, acute myeloid leukaemia; ALL, acute lymphoblastic leukaemia; CLL, chronic lymphocytic leukaemia; CML, chronic myeloid leukaemia; DLBCL, diffuse large B-cell lymphoma; MSCs, mesenchymal stromal cells.

#### 4.1.1 Transient receptor potential channels

It is thought that the primary physiological roles of TRP channels are perception of various sensations ranging from pain, pressure, temperature, taste and vision. However, evidence accumulates that TRP channels also regulate proliferation, differentiation, invasion, metastasis, autophagy and apoptosis of malignant cells (80, 81, 166–170). TRP channels have been shown to contribute crucial oncogenic functions in a number of hematologic malignancies (166, 167). Leukemia, lymphoma, myeloma and Waldenström macroglobulinaemia patient cells and cell lines have altered expression of TRP channels that has been linked with changes in cell proliferation, cell death and cell migration (165–167) (**Table 1**).

TRPM2 is overexpressed in cells from patients with acute myeloid leukemia (AML) and in AML cell lines (e.g. Kasumi-1, U937, KG-1, MV-4-11, SKNO1, THP-1, MonoMac-6, AML-193, MOLM13 and SHSY5Y) (129). TRPM2 depletion in AML cells and xenograft mouse models has anti-leukemic effects. TRPV2, TRPM7 and TRPC1 have been studied in chronic myeloid leukemia (CML) cell lines (K-562, KU812, MOLM-6 and 32D-p210) (127, 146, 147, 171). The silencing of TRPV2 induces significant apoptosis in K-562 cells (127), while inhibition of TRPM7 reduces cell proliferation and increases differentiation (146). In BCR::ABL1-expressing murine myeloid progenitor cells (32D-p210), TRPC1 expression is reduced and may be one of the factors associated with SOCE reduction in these cells (171).

TRPV1, TRPV6 and TRPM2 contribute to the growth of cells derived from acute lymphoblastic leukemia (ALL) (142, 172). TRPV1 activation by resiniferatoxin (an analog of capsaicin, a vanilloid agonist) induces apoptosis, interferes with cell cycle progression and decreases proliferation in both Jurkat T-cells and patient-derived T-ALL lymphoblasts; however, the affect of resiniferatoxin on non-leukemic cells was not tested (172). TRPV6 is one of the necessary elements for migration and oncogenic signaling in Jurkat T-cells (142). TRPM2 is crucial for cell cycle arrest and decreases apoptosis of irradiated Jurkat T-cells and Bcl-2-overexpressing T-lymphoblasts (143).

In chronic lymphocytic leukemia (CLL) cells, patient-derived and the Jok-1 cell line, TRPC1 plays a role in promoting cell survival. It does so by contributing to the production of anti-inflammatory cytokines and the activation of mitogen-activated protein kinase (MAPK)/extracellular signal-regulated kinase (ERK) pathways triggered by CD5 activation (150, 168). TRPML2 is associated with the sensitivity of plasma cell myeloma cell lines to ibrutinib and/or bortezomib treatment. TRPML2 expression is low in ibrutinib-resistant U266 cells but high in ibrutinib-sensitive RPMI8226 cells (163). Upon TRPML2 RNA-silencing, RPMI8226 cells show worse response to ibrutinib than controls (163). These data raise the possibility that TRPML2 expression levels may help predict ibrutinib sensitivity in patients with myeloma (163).

Most recently, somatic mutations and copy number variations in *TRP* genes have been reported in 33 cancer types including hematologic malignancies, in particular diffuse large B-cell lymphoma (DLBCL) and AML cells (173). TRP mutations in the transmembrane regions were concluded to be likely deleterious and these genetic alterations were possibly linked to transcripitional deregulation of *TRP* genes and the consequent change in expression of TRP channels (173). The frequency of mutations in TRP channels was higher in DLBCL than in AML cells, with TRPM2, TRPM3 and TRPM6 showing the greatest mutation frequency (173). However, it is not clear what significance these genetic alterations have in the pathogenesis of cancer. Further work is required to uncover how these mutations contribute to cancer initiation and progression, and whether they can serve as markers for diagnosis, prognosis, or as treatment targets (173). *Ex vivo* studies with patient-derived cells demonstrate that targeting of TRP channels offers potential to inhibit malignant cell proliferation and improve chemotherapy effects (129, 172).

#### 4.1.2 Purinoreceptor channels

P2X receptors are a family of ATP-dependent cation channels that have seven members (P2X1–7). An increase in extracellular ATP, often due to damage to the plasma membrane or exocytosis of ATP-containing granules, is the principal physiological stimulus for P2X receptor activation (174). Altered expression or function of P2X7 has been reported in a number of hematologic cancers (175). P2X7 is upregulated in cells from patients with AML and CLL and downregulated in B-ALL (124). Reports have differed on whether P2X7 in CML cells is up- or down-regulated (121, 124). When P2X7 activation is prolonged, and the receptor is exposed to high ATP levels, P2X7 opens an unselective membrane macropore and can trigger cell death (175, 176). P2X7RB is a splice variant that is unable to form this macropore (176). Both full-length P2X7RA and truncated variant P2X7RB are overexpressed in AML cells; whereas in relapsed AML patients, P2X7RB is increased and P2X7RA is decreased (126). AML blasts with high levels of P2X7RB have higher viability and much lower Ca^2+^ uptake than those expressing high levels of P2X7RA (126). AML development is slower and overall survival is extended in mice transplanted with P2X7-null AML cells compared to mice transplanted with control AML cells (125). Ca^2+^ influx is decreased in murine P2X7-null leukemia-initiating cells (LICs) and bulk AML cells compared to wild-type. The transcription factor cAMP-response element binding protein (CREB), which is involved in calcium signaling, is decreased in P2X7-null LICs and upregulated in AML patients. When CREB is overexpressed in P2X7-null AML cells, the development of leukemia is similar to wild-type AML cells (125). These results suggest that CREB-mediated Ca^2+^ signaling is required for the leukemogenic activities of P2X7.

#### 4.1.3 Nicotinic acetylcholine receptor

Upon binding acetylcholine, nAChR channels assist the movement of cations into the cell, which causes membrane depolarization (177) and triggers the opening of voltage-gated Ca_v_ channels leading to Ca^2+^ influx (151). Homomeric α7-nAChRs are more permeable to Ca^2+^ and desensitize faster than heteromeric nAChRs (177). Primary CLL cells express α7-nAChR at a higher level than normal B-cells, and inhibiting α7-nAChRs in a range of leukemic cell lines reduces cell migration (151). Conversely, protein expression levels of α7-nAChRs in AML, CML and ALL patient peripheral blood or bone marrow-derived mononuclear cells was lower than in healthy subjects (178). Acetylcholine causes an increase in intracellular Ca^2+^ levels in CML-derived K-562 cells, and the α7-nAChR antagonist methyllycaconitine citrate inhibits K-562 cell proliferation as well as reduces the intracellular Ca^2+^ levels (177). The opposite was observed in Jurkat T-ALL cells, with methyllycaconitine causing intracellular Ca^2+^ levels to rise but this did not require extracellular Ca^2+^ (179).

#### 4.1.4 *N*-methyl-D-aspartate receptor

**Figure 3:**
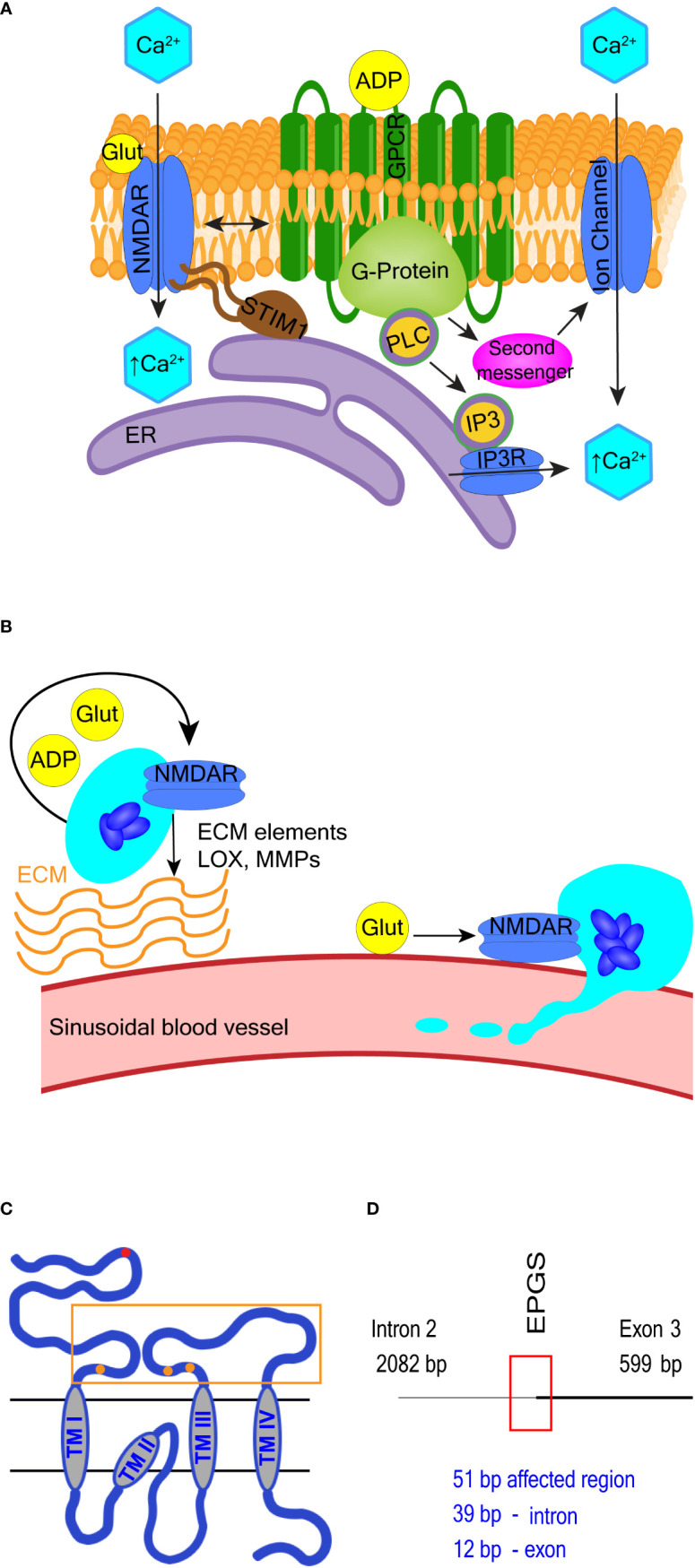
Selected NMDA receptor effects in hematopoietic cells. **(A)** Overview of NMDA receptor-induced calcium signaling. NMDA receptor directly facilitates Ca^2+^ entry into cells but may also operate in a metabotropic manner to induce Ca^2+^ release from the ER or *via* secondary messenger activation of ion channels such as transient receptor potential (TRP) channels. Adenosine diphosphate (ADP) and glutamate are both released from maturing megakaryocytes. ADP binds G protein-coupled receptors (GPCR) and activates PLC-β to increase intracellular Ca^2+^ levels. NMDA receptor modulates GPCR function in neuronal cells, so potentially may do so in hematopoietic cells. **(B)** Overview of NMDA receptor-associated effects in megakaryocytes. NMDA receptor assists proplatelet formation by regulating the expression of extracellular matrix (ECM) elements (e.g. collagen) and ECM remodeling enzymes (e.g. lysyl oxidase, LOX and matrix metalloproteinases, MMPs). **(C, D)** Schematics of NMDA receptor subunit GluN2C and the *GRIN2C* gene variants discovered in B-ALL. In **(C)**, the glutamate-binding domain (400-539; 659-800 aa) is enclosed in an orange rectangle, and glutamate binding sites are represented by orange dots (at 509-511, 516, 687-688, and 729 aa respectively). Location of *GRIN2C* variants found in B-ALL is marked by a red dot in **(C)** and red rectangle in **(D)** The affected region is 51 base pairs long; the EPGS sequence is translated (134-137 aa).

Typical neuronal NMDA receptors are ligand-gated non-specific cation channels with high Ca^2+^ permeability activated by glutamate and glycine (93). In non-neuronal cells, including in megakaryocytes, NMDA receptors may also function in a metabotropic-like (i.e. flux independent) manner (97, 98, 180, 181) (see **Figure 3A** and the corresponding legend for molecular details). In leukemic cell lines with megakaryocytic features Meg-01, K-562, and Set-2, NMDA receptor supports cell proliferation (182). Deletion of NMDA receptor in Meg-01 cells shifts cell differentiation toward the erythroid lineage, suggesting NMDA receptor function at the level of a bipotential megakaryocyte-erythroid progenitor (183). NMDA receptor inhibitor memantine enhances cytotoxic effects of cytarabine in Meg-01 cells, thus this drug combination warrants testing on patient cells (183). In non-leukemic mice, the NMDA receptor regulates proplatelet formation through a mechanism that involves megakaryocyte interaction with the extracellular matrix and cytoskeletal reorganization (180). NMDA receptor exerts these effects by influencing Ca^2+^ and adenosine diphosphate (ADP) signaling, and the expression of transcripts involved in extracellular matrix remodeling (180) (**Figure 3B**). These mechanisms are relevant to the pathophysiology of primary myelofibrosis (PMF); therefore, NMDA receptor inhibitors should be tested in PMF models.

In a random survival forest model, variants in the *GRIN2C* gene encoding the GluN2C subunit of NMDA receptor were part of a group of 7 variant genes found to predict shorter event-free survival in high-risk pediatric patients with B-ALL (145). The mutated *GRIN2C* region in ALL covers an intron/exon boundary located in the GluN2C protein’s N-terminal domain (see **Figures 3C, D** for further details). The presence of *GRIN2C* mutations was associated with accelerated relapse in children with high-risk B-ALL, but their functional impact is not known. These findings call for experimental studies to determine the NMDA receptor role in normal and leukemic B-cell precursors.

#### 4.1.5 Voltage-dependent anion channels

VDAC has three isoforms in mammals with VDAC1 being the most abundant (37, 104). VDAC1 is a key regulator of metabolite transfer between the mitochondria and cytosol including of ATP, ADP, and of small ions such as Ca^2+^ and Na^+^. These functions are crucial for normal mitochondrial bioenergetics (37, 184). In its open state, VDAC1 facilitates metabolite exchange but is lowly permeable to Ca^2+^. In contrast, in the “closed” state VDAC1 is highly permeable to Ca^2+^ providing a proapoptotic signal (37, 185).

VDAC1 is overexpressed in U266 myeloma cells, which together with CD45 expression enhances the cells sensitivity to apoptosis *via* mitochondrial pathways (186). VDAC1 is also overexpressed in CLL patient cells compared to healthy controls (187). VDAC1-derived decoy sequences (Antp-LP4 and N-terminal-Antp) induce cell death in peripheral blood mononuclear cells from patients with CLL but not from healthy donors (187). Similarly, in a large panel of leukemic cell lines including from CLL (MEC-1, MO1043, and CLL), T-ALL (MOLT4, Jurkat), and AML (U-937, THP-1, K-562), VDAC1-targeting peptides induce cell death (188).

VDAC1 associating with Bcl-2, Bcl-xL or hexokinase prevents apoptosis in cancer cells. VDAC1 peptides disrupt this association leading to VDAC1 oligomerization, mitochondrial Ca^2+^ overload, cytochrome c release and apoptosis (187, 188). Combined treatment of acute promyelocytic leukemia (APL) cell line HL-60 with melatonin and retinoic acid decreases VDAC1 expression, suggesting its leukemia-promoting role (189). In B-ALL cell lines, VDAC1 was upregulated after prednisolone treatment in three steroid-sensitive cell lines (697, Sup-B15, RS4;11) but unchanged in the steroid-resistant cell line (REH), suggesting that VDAC1 has a role in steroid-induced apoptosis (190). Similar was seen in T-ALL cells. Cell death occurred in Jurkat T-cells when either rice or human VDAC proteins were overexpressed, and the effect was blocked by ectopically expressed Bcl-2 (191). Overall, VDAC1 interactions with pro-survival proteins support anabolic metabolism and inhibit apoptosis thus maintaining leukemia growth. Strategies that target these interactions are being explored for treatment of leukemia, with T-ALL cells emerging as the most vulnerable to this form of manipulation (192, 193).

#### 4.1.6 Voltage-gated ion channels (Ca_V_ channels)

Voltage-gated Ca^2+^ channels are coded by *CACNA* genes (calcium voltage-gated channel subunit alpha) and are subdivided into three families Ca_V_1, Ca_V_2 and Ca_V_3 (194). Ca_V_1 and Ca_V_3 channels are expressed in many cell types while Ca_V_2 are mostly expressed in neurons (106). Ca_V_ channels mediate Ca^2+^ influx in response to plasma membrane depolarization, influencing muscle contraction and neurotransmission, as well as secretion and gene expression in may cell types (137). Ca_V_1 channels are activated by high voltage (> −40 mV with a peak at 0 mV) and mediate long-lasting (L-type) currents with slow inactivation. In contrast, Ca_V_3 channels are activated by low voltage (around −60 mV with a peak at −20 mV) and mediate transient currents (T-type) with faster kinetics than the L-type currents. Ca_V_2 channels are activated by high voltage and mediate P/Q-type, N-type and R-type Ca^2+^ currents (106).

Bioinformatic analysis of Oncomine, a web-based cancer microarray database of patient tissue revealed increased expression of *CACNA* transcripts in diverse cancer types including of *CACNA1S* and *CACNA1C* (coding for Ca_V_1.1 and Ca_V_1.2 channels respectively) in AML and B-ALL samples and of *CACNA1A* (coding for Ca_V_2.1) in samples from patients with CLL, marginal zone lymphoma and monoclonal gammopathy of unknown significance (136, 137). On the other hand, downregulation of *CACNA1C* transcripts (coding for Ca_V_1.2) was seen in centroblastic lymphoma, *CACNA1F* (coding for Ca_V_1.4) in anaplastic large cell lymphoma, and *CACNA1G* (coding for Ca_V_3.1) in mantle cell lymphoma (195). A recent study confirmed distinct expression of Ca_V_ channels in a range of lymphoma cell lines and patient-derived samples (156). Ca_V_1.2 (*CACNA1C*) expression was increased in classical Hodgkin lymphoma cell lines when compared to other B-cell lymphoma cell lines. Ca_V_1.3 (*CACNA1D*) showed higher expression in samples from patients with activated B-cell-like DLBCL (ABC-DLBCL), whereas expression of Ca_V_1.1 (*CACNA1S*), Ca_V_1.2 (*CACNA1C*), and Ca_V_1.4 (*CACNA1F*) were higher in germinal centre B-cell like DLBCL (GCB-DLBCL) (156). Therapeutic potential of inhibiting Ca_V_1.2 in AML was revealed in an elegant 3D-culture model that mimicked the human bone marrow niche and utilized AML-derived mesenchymal stromal cells (AML-MSCs) from pediatric patients (138). Inhibition of Ca_V_1.2 channel in AML-MSCs using lercanidipine (an anti-hypertensive drug) interfered with leukemia growth *ex vivo* and *in vivo*, with no toxic effects on normal MSCs or healthy CD34-positive HSCs (138). Further work is required to determine the mechanism through which Ca_V_ channels influence blood cancer growth.

#### 4.1.7 Orai1 channels

Multimers of Orai1 proteins form an ion pore in the plasma membrane that is highly selective for Ca^2+^. SOCE is triggered when Orai1 and STIM1/STIM2 proteins interact in response to ER Ca^2+^ store depletion. Increased expression of Orai1 or STIM1/STIM2 has been recorded in cell lines derived from AML (132, 133), T-ALL (134), CLL (11) and various lymphoma cell lines (134, 156). Mantle cell lymphoma Rec-1 cell line does not have high expression of Orai1 and STIM1 but Rec-1 and patient cells have significantly higher cytoplasmic Ca^2+^ concentrations under physiological conditions compared to normal cells suggesting “leaky SOCE” (196). High expression of Orai1 and STIM1 in CLL patients is associated with worse treatment- and progression-free survival (11). In mice models of T-ALL, deletion of STIM1 and STIM2 abolishes SOCE and results in prolonged survival (134). The underlying mechanism is intriguing, as the absence of SOCE does not change leukemic cells proliferation; instead, prolonged survival is associated with reduced inflammation in organs infiltrated by leukemia (134). In the HL-60 APL cell line, silencing of Orai1 and Orai2 reduces cell migration and proliferation (132). In the KG-1 and U937 AML cell lines, Orai1 contributes to cell proliferation and cell cycle progression (133). *ORAI1* gene expression was increased in peripheral blood mononuclear cells from 9 patients with AML compared with normal cells and correlated with adverse risk in the cohort of 439 AML patients (133).

Orai1 and STIM1 function is also linked with the CD20 molecule in B-cells and required for the efficacy of anti-CD20 antibody therapy in B-cell cancers. CD20 (MS4A1) is part of the membrane-spanning 4-domain family, subfamily A (MS4A) (197). The exact biological role of CD20 is unknown but it may act as an amplifier of Ca^2+^ signals transmitted through the B-cell receptor (BCR) in immature and mature B-cells to modulate cell proliferation and differentiation (198). CD20 has been reported to be physically coupled to or affect the phosphorylation of BCR and BCR-associated kinases; which are upstream regulators of the signaling cascade that activates SOCE (199–201).

**Figure 4:**
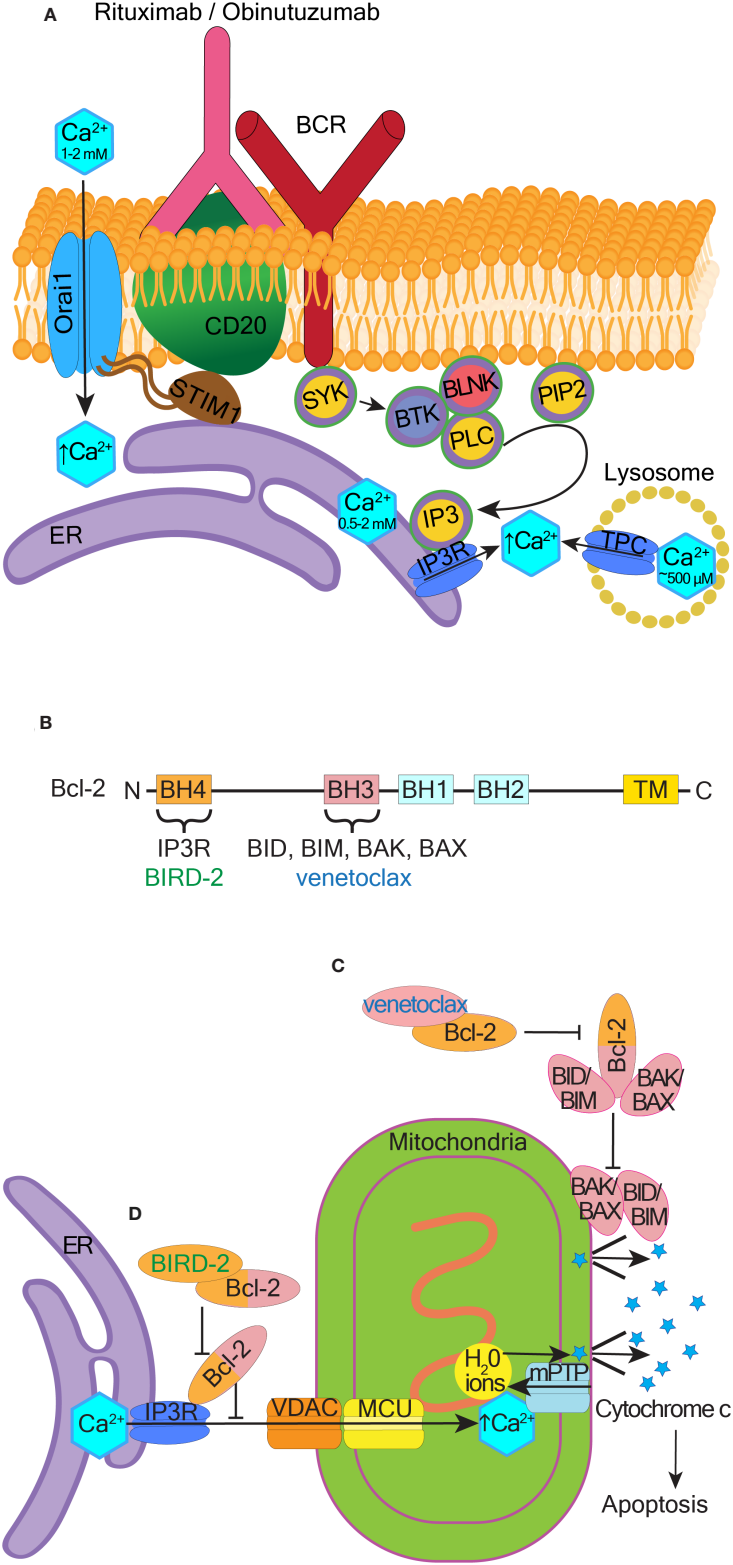
Therapeutic potential of pro-apoptotic calcium signaling at the ER and mitochondria. **(A)** Mechanism of BCR activated Ca^2+^ influx in response to rituximab and obinutuzumab. The membrane-spanning 4-domain protein CD20 is physically-coupled to the BCR. Rituximab and obinutuzumab induce phosphorylation of several proteins involved in BCR signaling, including BLNK (B-cell linker kinase), BTK (Bruton’s tyrosine kinase), and PLC-γ (phospholipase C-γ). CD20 binds STIM1 and this binding is dependent on the presence of Orai1. Upon binding of rituximab/obinutuzumab, Ca^2+^ is released from lysosomes, the ER, and/or extracellularly *via* activation of store-operated calcium entry, which assists cell killing. **(B)** Schematic showing the four Bcl-2 homology (BH) domains. Venetoclax binds to the hydrophobic cleft located in the BH3 domain, and BIRD-2 binds to the BH4 domain. Transmembrane domain (TM), N- and C- termini are indicated. **(C)** Canonical BAX and BAK dependent pathway of apoptosis and the mechanism through which venetoclax inhibits this pathway. **(D)** Non-canonical ER Ca^2+^-dependent pathway of apoptosis and the mechanism through which BIRD-2 inhibits this pathway.

Monoclonal anti-CD20 antibodies such as rituximab and obinutuzumab activate Ca^2+^ influx in patient CLL cells and cell lines such as SUDHL-4, BL2, Ramos, BL60, Raji, Daudi, and normal B-cells (202–206). Using genetically encoded Ca^2+^ indicator GCaMP-CD20 as a precise method to measure Ca^2+^ concentration changes around CD20, it was determined that anti-CD20 antibodies do not cause Ca^2+^ influx through or near CD20 (207). Instead, obinutuzumab activates intracellular Ca^2+^ efflux from either lysosomes or the ER into the cytosol (206) (see **Figure 4A** and the corresponding legend for molecular details). Inhibition of this intracellular Ca^2+^ movement reduces obinutuzumab-induced cell death (206, 207). Binding of rituximab to CD20 induces co-clustering of CD20 with Orai1 and STIM1 in SUDHL-4 cells, leading to extracellular Ca^2+^ influx and internal Ca^2+^ store release (205). CD20 overexpression in HEK293 cells increases Ca^2+^ influx, which is abolished when Orai1 and STIM1 are knocked down (207). CD20 strongly interacts with STIM1 but only when Orai1 is present (207). Influx of Ca^2+^ induced by rituximab or obinutuzumab is significantly reduced in Orai1 knockdown cells (205, 206). In B-CLL cells expressing high levels of STIM1, a combination of an anti-STIM1 monoclonal antibody and rituximab significantly reduces cell viability compared to rituximab alone (11). Thus, CD20 interactions with Orai1/STIM1 are important for the therapeutic efficacy of anti-CD20 antibodies. Manipulation of these interactions may help develop more effective therapeutic combinations for B-cell malignancies (**Figure 4A**).

### 4.2 IP3 signaling cascade and Ca^2+^ release from the ER

IP3 is a major regulator of Ca^2+^ signaling; it binds to IP3Rs on the ER to release Ca^2+^ into the cytosol (27, 28) (**Figure 1**). IP3 and another second messenger DAG are generated when PLC, activated downstream of G-protein coupled or tyrosine kinase receptors, hydrolyses PIP2 located in the plasma membrane (26).

#### 4.2.1 Phospholipase C

PLC has six isoforms; β, γ, δ, ϵ, ζ, and η. PLC-β operates downstream of GPCRs and PLC-γ downstream of tyrosine kinase receptors (29, 30). Important activators of PLC-γ in hematologic cells are BCR and T-cell receptors (TCR), which are transmembrane tyrosine kinase receptors (208). T-cells mainly express the PLC-γ1 isoform, and B-cells mainly express the PLC-γ2 isoform. PLC-γ1 is essential for IP3 production and Ca^2+^ release in normal T-cells, whereas PLC-β3 is the main regulator of these responses in Jurkat T-cells and patient-derived T-ALL blasts (209).

Leukemic stem cells (LSCs) are multipotent, proliferative, and self-renewing cells that propagate leukemia (210). In AML LSCs, oxysterol-binding protein-related protein 4L (ORP4L) acts as a scaffold protein that facilitates PIP2 presentation to PLC-β3 for cleavage into IP3 (211). ORP4L is expressed in LSCs, but not in normal HSCs (211). Knocking down or inhibiting ORP4L decreases the survival of T-ALL cells and AML LSCs and reduces spontaneous cytosolic and mitochondrial Ca^2+^ oscillations (209, 211, 212). In T-ALL cells, ORP4L also interacts with PLC-β3 and IP3R1, which enhances Ca^2+^ release from the ER by facilitating the binding of IP3 to IP3R1 (212). Overall, the ORP4L regulated Ca^2+^ release into the mitochondria helps sustain mitochondrial oxidative respiration required for survival of T-ALL cells and AML LSCs (209, 211, 212)

BCR and TCR recruit kinases such as SYK (spleen tyrosine kinase), BTK (Bruton’s tyrosine kinase), BLNK (B-cell linker kinase), and ZAP70 (zeta chain of T-cell receptor-associated protein kinase 7) to phosphorylate and activate PLC-γ. PLC-γ2 plays a role in CLL, DLBCL, Hodgkin lymphoma, endemic Burkitt lymphoma, MALT-associated gastric lymphoma, and plasma cell myeloma (208). For example, B-CLL cells showing high responsiveness after BCR engagement have higher PLC-γ2 activity and calcium signaling compared to non-responding cells (213). Patients with such hyperresponsive B-cells have a poorer prognosis than non-responders (213). Ibrutinib, which inhibits BTK and thus blocks PLC-γ2 signaling, has become an important and effective treatment for CLL and other B-cell lymphomas (208).

#### 4.2.2 Inositol 1,4,5-trisphosphate receptors

Three isoforms of IP3Rs exist and most cell types express more than one isoform (214). Mice with all three IP3R isoforms deleted develop T-cell malignancies throughout the body that resemble T-ALL (215). Cytogenetically normal AML cells have higher expression of IP3R2 than healthy progenitors and patients with high IP3R2 expression have shorter overall and event-free survival (135). DLBCL SU-DHL-4 cells also have high levels of IP3R2 and constitutive IP3 signaling, which leads to elevated basal levels of cytoplasmic Ca^2+^ and increased cell survival (154). Inhibition of IP3 production *via* inhibiting PLC reverses the prosurvival effect and increases cell death in SU-DHL-4 cells (154).

Several oncogenes and tumor suppressors directly interact with IP3Rs and regulate their activities to control Ca^2+^ influx into the mitochondria. Among such IP3R regulators are the Bcl-2 family proteins, which consist of different anti-apoptotic members (e.g. Bcl-2, Bcl-xL, Mcl-1, and Bcl-10) and pro-apoptotic members (e.g. BIM, BID, BAX, and BAK) (216) (**Figure 4B**). Bcl-2 overexpression is common in blood cancer, including in DLBCL, AML and CLL (154, 217, 218). Bcl-2 binds to and prevents the activation of pro-apoptotic proteins through their BH3 domains (219, 220). To overcome Bcl-2 effects in cancer cells, BH3 mimetics like venetoclax were developed that target the hydrophobic BH3-binding groove of Bcl-2 (**Figure 4B**). Venetoclax binding to Bcl-2 liberates BIM, which activates BAX or BAK, leading to apoptosis (221) (**Figure 4C**).

In addition to the canonical BAX/BAK-dependent pathway of apoptosis, Bcl-2 also directly binds to IP3Rs on the ER through its BH4 domain. The binding of Bcl-2 to IP3R inhibits Ca^2+^ release from the ER and prevents cell apoptosis triggered by mitochondrial Ca^2+^ overload (222). Venetoclax does not interfere with this BH4-dependent mechanism of cell death (223). In contrast, a designer peptide Bcl-2 IP3R Disruptor-2 (BIRD-2) targets the BH4 domain of Bcl-2 (224) (**Figure 4B**). BIRD-2 binding to Bcl-2 unleashes IP3R activation and cytotoxic Ca^2+^ levels are released from the ER (155, 225) (**Figure 4D**).

BIRD-2 induces apoptosis in multiple blood cancer cell lines and/or patient-derived cells, including from DLBCL, CLL, plasma cell myeloma, and follicular lymphoma (153, 226, 227). DLBCL cells with high levels of IP3R2 and constitutive IP3 signaling are particularly sensitive to BIRD-2 (154). In DLBCL cell lines, BIRD-2 induces cell death in a caspase-dependent manner, however in contrast to venetoclax, BIRD-2-induced cell death is independent of BAX/BAK (228). In both DLBCL cell lines and primary CLL patient samples, BIRD-2 triggers mitochondrial Ca^2+^ overload to induce caspase-dependent cell death. Cyclosporine A, which desensitizes mPTP to excessive Ca^2+^-induced opening, and ruthenium265 (Ru265), which inhibits mitochondrial Ca^2+^ uptake, both counteract BIRD-2-induced cell death (228). Combining venetoclax with BIRD-2 enhances cell death of DLBCL cell lines, however DLBCL cells with acquired resistance to venetoclax were not sensitized to BIRD-2 (155, 229). BIRD-2 may be useful in combination with venetoclax as a therapeutic strategy in DLBCL, however the clinical relevance of these approaches remains to be determined.

Another therapeutic target that can induce mitochondrial Ca^2+^ overload is the GPCR neurokinin-1 receptor (NK-R1), expression of which is elevated in patient-derived AML cells and cell lines (230). Targeting NK-R1 with the antagonists SR140333 or aprepitant in AML and CML cell lines increases cytosolic and mitochondrial Ca^2+^ concentrations, resulting in increased production of reactive oxygen specied (ROS) and apoptosis (230). When IP3R or VDAC1 are inhibited, ROS production and apoptosis are decreased (230), but neither antagonist inhibits proliferation of normal CD34-positive HSCs. Aprepitant has been approved by the US Food and Drug Administration for the treatment of chemotherapy-induced nausea and vomiting (230). Therefore, this and other NK-R1 antagonists could be repurposed for testing efficacy in AML (230).

### 4.3 Endo-lysosomal Ca^2+^ channels

The role of endo-lysosomal Ca^2+^ signaling in blood cancer has not been systematically studied but there are a number of observations pointing towards its importance. TRPML3, TPC1 and TPC2 endo-lysosomal Ca^2+^ efflux channels are expressed in the megakaryoblastic leukemia cell line Meg-01 (231). NAADP releases Ca^2+^ from the lysosomal-like Ca^2+^ stores in Meg-01 cells and TPC knockdown reduces this response (231). TPC2 is localized to platelet dense granules that are lysosome-related organelles and is involved in their maturation and function (232). TPC2 mediates Ca^2+^ release and formation of perigranular Ca^2+^ nanodomains in Meg-01 cells that mark “kiss-and-run” events mediating material transfer between different granules (232). Upon genetic deletion of NMDA receptors in Meg-01 cells, accumulation of lysosomal organelles and upregulation of *MCOLN3* transcripts (coding for TRPML3) were observed. This suggests a link between lysosomal biogenesis, NMDA receptor function and Meg-01 cell proliferation (183). TPC1 and TPC2 inhibitor tetrandrine suppresses growth and increases cell death in several AML cell lines (U937, NB4, K-562, HL-60 and THP-1) (233–236). A recent study also demonstrates that TPC2 inhibition and its genetic deletion sensitizes ALL cells (cell lines and patient-derived) to cytotoxic drugs (237). Upon TPC2 deletion, leukemic cells are not able to sequester cytotoxic drugs within lysosomes, which increases drug concentration in the cytoplasm and enhances its cytotoxic effectiveness. Therefore, targeting lysosomal TPC2 may help overcome chemoresistance in ALL cells (237). Similar may apply in AML, although different mechanisms may contribute to lysosomal deregulation in different types of leukemia (238–240).

### 4.4 Calcium ATPases and secondary-active Ca^2+^ transporters

Several types of Ca^2+^ ATPases are involved in the maintenance of transmembrane Ca^2+^ gradients between the cytosol and the blood plasma as well as between the cytosol and the inner compartments of the organelles. Plasma membranes are equipped with several isoforms of the plasma membrane calcium ATPases (PMCAs) (241, 242) (**Figure 1**). Human monocytes and macrophages express plasma membrane Na^+^/Ca^2+^ exchanger NCX that actively extrudes Ca^2+^ while taking in Na^+^ transported passively using the energy of transmembrane Na^+^ gradient generated by the Na^+^/K^+^ ATPase (243). Mitochondrial membrane contains its own Na^+^(Li^+^)/Ca^2+^ exchanger (NCLX) that controls Ca^2+^ levels in the mitochondria (244). Cells also pump Ca^2+^ out of the cytoplasm into the Golgi apparatus through secretory pathway Ca^2+^ ATPases (SPCA), and to the ER through SERCA (245) (**Figure 1**). The activity of SERCA reflects the activation state of T-cells (246), B-cells (247), Th1 and Th2 lymphocytes (248). Of these molecules, SERCA has been reported to be dysregulated in a number of hematologic malignancies.

#### 4.4.1 Sarco-endoplasmic reticulum calcium ATPase

There are three SERCA genes in humans and alternative splicing can give rise to many isoforms (10). In response to differentiation, SERCA3 expression changes in leukemic megakaryocytic cell line Meg-01, precursor B-ALL cell lines (Kasumi-2 and RCH-ACV), and APL cell lines (NB4 and HL-60) (249–251). All-trans retinoic acid-induced differentiation of APL cells results in increasing SERCA3 expression and SERCA3-dependent Ca^2+^ accumulation (249). When SERCA activity is inhibited, lower concentrations of retinoic acid can induce differentiation of NB4 and HL-60 cell lines (252).

In a human T-ALL xenograft mouse model, SERCA inhibition with thapsigargin reduces tumor growth (253). Thapsigargin prevents the activation of the transmembrane receptor, NOTCH1, which often contains activating mutations in T-ALL, CLL, mantle cell lymphoma, and a subset of DLBCL (10, 253). The reduction of Ca^2+^ entering the ER upon SERCA inhibition changes the folding and trafficking of NOTCH1 (10, 253). As reviewed by Pagliaro et al, other SERCA inhibitors have been developed that induce apoptosis in a range of leukemic cell lines and xenograft models including T-ALL, B-ALL, mantle cell lymphoma, and AML (10). In contrast, when SERCA expression is reduced or its activity is inhibited by oncogenes such as Bcl-2 and Ras, the decrease in ER Ca^2+^ concentrations reduces the potential for a Ca^2+^ overload and initiation of apoptosis in response to ER stress (219, 254).

### 4.5 Calcium sensor and effector proteins

Some of the downstream Ca^2+^ sensor proteins implicated in blood cancer include the S100 family, calcium/calmodulin-stimulated protein kinases (CaMKs), calcineurin, CALR, and PKC. Dysregulation of these Ca^2+^ sensors alters gene regulation associated with cell apoptosis, proliferation, and migration (**Table 2**).

**Table 2:** The differential expression of calcium sensor and effector proteins and their relative contribution to the malignant phenotype of different blood cancers.

Cancer type	Molecule	Change in disease	Functional effects	References
AML	S100-A4	↑ expression	↓ overall survival ^(P)^, ↑ cell proliferation ^(C)^, ↑ migration ^(C)^, ↑d rug resistance ^(C)^	(255–259)
	S100-A8 or -A9	↑ expression	↓ overall survival ^(P,M)^, ↓ event free survival ^(P)^, ↑ drug resistance ^(C)^, ↑ autophagy ^(C)^, ↓ apoptosis ^(C)^, differentiation ^(C,P,M)^	(255, 260–265)
	S100-P	↑ expression	↑ overall survival ^(P)^, differentiation ^(C)^	(259, 266–268)
	CAMKI	↑ expression	↑ cell proliferation ^(C,M)^, ↓ overall survival ^(P)^	(269)
	CAMKII	↑ activation	↓ differentiation ^(C)^, ↑ cell proliferation ^(C)^	(270)
	CAMKIV	↑ expression	↑ cell proliferation ^(C,M)^, ↓ overall survival ^(P)^	(269)
	PKCα	↑ expression and activation	↑ Bcl-2 phosphorylation ^(P)^, ↓ overall survival ^(P)^	(271–273)
ALL	S100-A4	↑ expression ^(C,P)^		(258, 274)
	S100-A6	↑ expression	↓ overall survival ^(M)^, ↑ cell proliferation ^(M)^, ↓ apoptosis ^(C)^	(274–278)
	S100-A8 or A9	↑ expression	↓ event free survival ^(P)^, ↓ Ca^2+^ influx ^(C)^, ↑ drug resistance ^(C,P)^, ↑ relapse ^(P)^	(274, 279)
	Calcineurin	↑ activation	↑ cell proliferation ^(C,M)^, ↓ apoptosis ^(C,M)^, ↑ disease progression ^(M)^	(280, 281)
CML	S100-A4	↑ expression	↑ drug resistance ^(C)^	(282)
	S100-A8 or A9	↑ expression	↑ drug resistance ^(C)^, ↑autophagy ^(C)^, ↓ Ca^2+^ influx ^(C)^	(260, 283)
	CAMKII	↑ activation	↑ cell proliferation ^(C)^	(270)
	CAMKII	↑ expression	↑ drug resistance ^(P)^	(284)
	PKCα	↓ expression	↑ association with cell membrane ^(P)^	(285)
	PKCβ2	↑ expression ^(P)^		(286)
MPN	S100-A4	↑ expression	↑ inflammation ^(P)^	(287)
	S100-A8 or A9	↑ expression	↑ inflammation ^(P)^, ↓ cell proliferation ^(P)^	(287)
	FKBP5	↑ expression in megakaryocytes	↑ cell survival ^(P),^ ↓ calcineurin activity ^(P),^ ↑ STAT5 activation ^(C,P)^	(288, 289)
	CALR	mutations, predicted loss of Ca^2+^ binding	↑ Ca^2+^ influx ^(P)^, ↑ proliferation of megakaryocytes ^(P)^	(9, 290, 291)
CLL	S100-A8	↑ expression	↑ disease progression ^(P)^, ↑ need for early treatment ^(P)^	(292)
	PKCα	↓ expression ^(P)^		(286)
	PKCβ2	↑ expression, ↑ activation	↓ Ca^2+^ influx ^(P)^, ↑ cell survival ^(P,M)^, ↓ apoptosis ^(P)^	(286, 293, 294)
DLBCL	S100-A4	↑ expression	↑ drug resistance ^(P)^	(295)
	S100-A8 or A9	↑ expression	↑ drug resistance ^(P)^	(295)
	Calcineurin	↑ activation	↑ cell proliferation ^(C)^, ↓ apoptosis ^(C)^	(280, 296)
T-cell lymphoma	S100-A9	↑ expression	↑ drug resistance ^(P)^, ↓ overall survival ^(P)^, ↓ progression free survival ^(P)^, ↑early reoccurrence rate ^(P)^	(297, 298)
Plasma cell myeloma	CAMKII	↑ expression	↑ disease progression ^(P)^, ↓ overall survival ^(P)^, ↑ cell proliferation ^(C)^, ↓ apoptosis ^(C)^	(299)

(P) = Patient cells, (C) = Cell lines, (M) = Mouse model. ↑ = increased, ↓ = decreased. Expression changes are often found in particular cell lines or leukemic subtypes and not in others. Empty cells indicate there is no data. AML, acute myeloid leukemia; ALL, acute lymphoblastic leukemia; CLL, chronic lymphocytic leukemia; CML, chronic myeloid leukemia; DLBCL, diffuse large B-cell lymphoma; MPN, myeloproliferative neoplasms (classical Philadelphia chromosome-negative).

#### 4.5.1 S100 family

The S100 family are Ca^2+^ binding proteins of which many have been reported to be dysregulated in AML, ALL, CLL, MPN, and lymphomas (292, 295, 300, 301). S100- A8 and A9 are the most well-studied members of the S100 family in leukemia. Dysregulation of their expression, and changes in plasma levels, or secretion levels in the bone marrow microenvironment have been reported in AML (300, 301). S100- A8 and A9 are dose-dependent regulators of myeloid differentiation and leukemic cell proliferation and can play contradictory roles, depending on their expression levels as monomers, homodimers, or heterodimers (300, 301). S100- A8 and A9 are expressed constitutively in the cytoplasm of myeloid cells, including myeloid precursors, but are absent from lymphocytes (301). Increased activity of multiple S100 family members is associated with increased drug resistance in hematologic malignancies including AML, CML, ALL, and B-cell lymphomas (302, 303). For example, S100-A8/A9 contribute to gilteritinib resistance in FLT3- internal tandem duplications- (FLT3-ITD)-positive AML primary cells and cell lines (304). Particularly, S100-A9 expression has been found to be more consistently and remarkably altered than S100-A8 in human FLT3-ITD-positive AML cell lines (MOLM13 and MOLM13-RES) after gilteritinib treatment. The potential mechanism is gilteritinib promotes Bcl-6 dissociation from the S100-A9 promoter, which leads to upregulation of S100-A9 (304).

#### 4.5.2 Calcium/calmodulin-stimulated protein kinase family

Calmodulin is a Ca^2+^-binding protein that regulates a wide variety of cellular processes *via* interaction with multiple target proteins (305). The CaMK family members are activated when bound to Ca^2+^-saturated calmodulin (306). CaMK family members are overexpressed or aberrantly activated in CML, AML, and plasma cell myeloma (269, 270, 284, 299, 307). Inhibition or knockdown of CaMKI, CaMKII, or CaMKIV reduces proliferation in different myeloid leukemia cells and multiple CAMK or calmodulin antagonists have been used to inhibit leukemic cell growth and proliferation (269, 270, 305–307). High expression of CaMKs is associated with a poor overall survival probability in patients with plasma cell myeloma or AML (269, 299). Deletion of CaMKII suppresses T-cell lymphomagenesis in mice, and T-cell lymphoma cell line growth (comprising H9, JB6, Jurkat, and SU-DHL-1) is suppressed when CaMKII activity is inhibited (308). CaMKII is also activated by the constitutively active tyrosine kinase BCR::ABL1 in CML cells. The tyrosine kinase inhibitor (TKI) imatinib, inhibits proliferation of BCR::ABL1 expressing cells and is accompanied by a rapid decrease in activated (autophosphorylated) CaMKII (270). In an inducible BCR::ABL1 cell line (TonB210.1), decreased BCR::ABL1 expression is also accompanied by a reduction of autophosphorylated CaMKII, and inducing BCR::ABL1 expression restores CaMKII autophosphorylation (270). CAMKII-γ is highly activated in CML LSCs and its aberrant activation accelerates CML blast crisis (309). In a mouse xenograft model of patient-derived CML cells, LSCs were eliminated by an ATP-competitive CAMKII-γ inhibitor berbamine (310).

#### 4.5.3 Calcineurin

**Figure 5:**
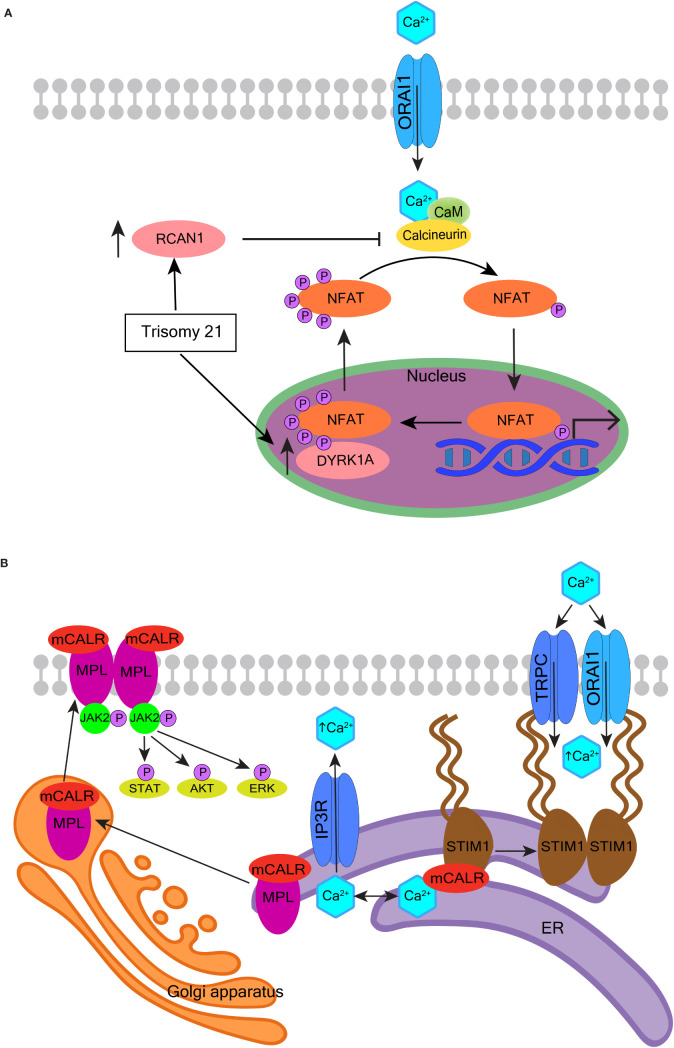
Oncogenic effects of calcineurin and calreticulin. **(A)** The role of calcineurin-NFAT signaling in the pathogenesis of myeloid proliferation associated with Down syndrome. Human chromosome 21 encodes two important regulators of nuclear factor of activated T-cells (NFAT) - regulator of calcineurin 1 (RCAN1) and dual specificity tyrosine phosphorylation regulated kinase 1A (DYRK1A). When calcineurin is activated by Ca^2+^ and calmodulin (CaM), it dephosphorylates NFAT. Dephosphorylated NFAT translocates to the nucleus and transcriptionally regulates numerous genes involved in cell proliferation, growth, migration, differentiation, and survival. NFAT is re-phosphorylated by DYRK1A and is exported back to the cytoplasm. RCAN1 inhibits calcineurin, and so also inhibits the dephosphorylation and translocation of NFAT. RCAN1 and DYRK1A are overexpressed in Down syndrome and are suspected to contribute to the development of transient abnormal myeloproliferation and megakaryoblastic leukemia in Down syndrome children. **(B)** The role of mutated calreticulin (CALR) in myeloproliferative neoplasms. More than 50 mutations have been reported in exon 9 of the *CALR* gene; most generate a +1-frameshift causing the mutated CALR protein (mCALR) to stably associate with the thrombopoietin receptor MPL protein in the ER. The mCALR-MPL complex is transported from the ER through the Golgi apparatus and secretory system to the plasma membrane. The binding of mCALR to MPL constitutively activates signaling through JAK2 and its downstream targets such as STAT, AKT, and ERK (left). The most common mCALR variants are type 1 that also impair the Ca^2+^ binding activity of mCALR more than type 2. The type 1 mCALR with reduced Ca^2+^ binding dissociates from STIM1 in the ER. This allows STIM1 to dimerize and bind Orai1 and TRPC, which leads to constitutive activation of SOCE (right).

Calcineurin is a calcium-calmodulin-dependent phosphatase, that when activated by Ca^2+^ and calmodulin, dephosphorylates its substrates including nuclear factor of activated T-cells (NFAT) (311). Dephosphorylated NFAT proteins translocate into the nucleus to regulate the transcription of genes important for cell proliferation, growth, migration, differentiation, and survival (311). The calcineurin-NFAT pathway negatively regulates megakaryopoiesis (312). Inappropriate inhibition of this pathway may contribute to the pathological expansion of megakaryocytes and their precursors, in particular in the context of Down syndrome (313, 314) (see **Figure 5A** and the corresponding legend for molecular details). The calcineurin inhibitor FKBP5 (FK506 binding protein) is overexpressed in megakaryocytes from patients with PMF. FKBP5 overexpression in UT-7 cells (a factor-dependent human cell line with a megakaryocytic phenotype) promotes cell survival after cytokine deprivation, suggesting a pathway to disease development through the inhibition of calcineurin (288).

Calcineurin and NFAT have also been implicated in other hematologic malignancies, including Burkitt lymphoma, T-cell lymphoma, T-ALL, DLBCL, CML, CLL, and AML (311, 315, 316). The calcineurin inhibitors, cyclosporin A, and tacrolimus (FK506), have anti-leukemic effects in mice T-ALL models, and deletion of calcineurin specifically in T-ALL leukemic cells results in impaired leukemia progression (280, 281). Inhibition of calcineurin by cyclosporin A or FK506 is also selectively cytotoxic against the ABC-DLBCL (296). This response to calcineurin inhibitors is associated with reduced NFAT-mediated expression of critical genes, including c-Jun, signal transducer and activator of transcription 3 (STAT3), interleukin-6 and interleukin-10 that are crucial for survival of ABC-DLBCL cells (296).

#### 4.5.4 Calreticulin

CALR, amongst its other functions, is an ER-resident Ca^2+^-buffering protein that helps maintain intracellular Ca^2+^ homeostasis and assists the folding of proteins destined for secretion or insertion into the plasma membrane (317). CALR is mutated in approximately one-quarter of the Philadelphia chromosome-negative MPNs, PMF and essential thrombocythemia (290, 318). The mutations in *CALR*, as well as *JAK2* and *MPL* (**Figure 5B**, left), converge to constitutively activate JAK2-STAT signaling, which drives deregulated expansion of HSCs and megakaryocytes (317, 318). *CALR* mutations have two main variants, type 1 and type 2. Type 1 or type 1-like mutations are mostly large deletions of which a 52-bp deletion is the most common; while type 2 or type 2-like are mostly small insertions of which a 5-bp insertion is the most common. Type 1 mutations are predicted to impair the Ca^2+^-binding activity of CALR more than type 2 (319). Congruently, type 1 mutations associate with higher ER Ca^2+^ release, higher SOCE, and spontaneous cytosolic Ca^2+^ oscillations in cultured megakaryocytes (9, 290).

CALR assists the folding of STIM1, and whilst they are bound, STIM1 is in an inactive configuration on the ER membrane inhibiting SOCE (9). Megakaryocytes with mutated CALR have a decreased association between CALR and STIM1 (**Figure 5B**, right), and the CALR type 1 variant has the highest level of dissociation from STIM1. Defective interaction between mutant CALR and STIM1 activates SOCE and generates spontaneous cytosolic Ca^2+^ influx. This, in turn, increases megakaryocyte proliferation, which can be reversed using a specific SOCE inhibitor (9). Thus, the impact of mutated CALR on Ca^2+^ homeostasis may be influencing the course of MPN in combination with its aberrant activation of JAK2-STAT signaling. Further elucidation of these mechanisms may inform the development of new drugs to improve the effects of JAK2 inhibition.

#### 4.5.5 Protein kinase C

PKC is activated by the second messenger DAG, which is hydrolyzed from PIP2 following receptor engagement and PLC activation (320, 321). The PKC family has many isoforms that can be categorized into three groups: conventional PKC isoforms, novel PKC isoforms, and atypical PKC isoforms (320, 321). A range of PKC isoforms are up- or down-regulated which can affect cell growth and survival in AML, CML, CLL, and plasma cell myeloma (286, 322, 323). Only the conventional PKC isoforms (α, β, and γ) are activated by Ca^2+^ as well as DAG (320, 321). In CML, the BCR::ABL1 phosphorylates a range of PKC isoforms leading to altered activity (323). ER Ca^2+^ release and SOCE are reduced in cell lines that express BCR::ABL1 (171, 324). These abnormal Ca^2+^ responses are Bcl-2 independent but PKC dependent. PKC-β overexpression is significantly associated with resistance to TKIs such as imatinib (323, 325). Suppressing PKC-β activity or expression in TKI-resistant CML patient cells and cell lines increases the sensitivity to imatinib (325). Inhibition of PKC-β increases the effect of imatinib on reducing leukemic cell proliferation in a CML mouse model and also prolongs survival (325). Outside of CML, PKC-β was found to be essential for CLL development in a mouse model and promotes cell growth and survival of CLL cells (294, 322, 326).

## 5 Mutational landscape in the calcium-toolkit encoding genes recorded in publically accessible blood cancers databases

**Figure 6:**
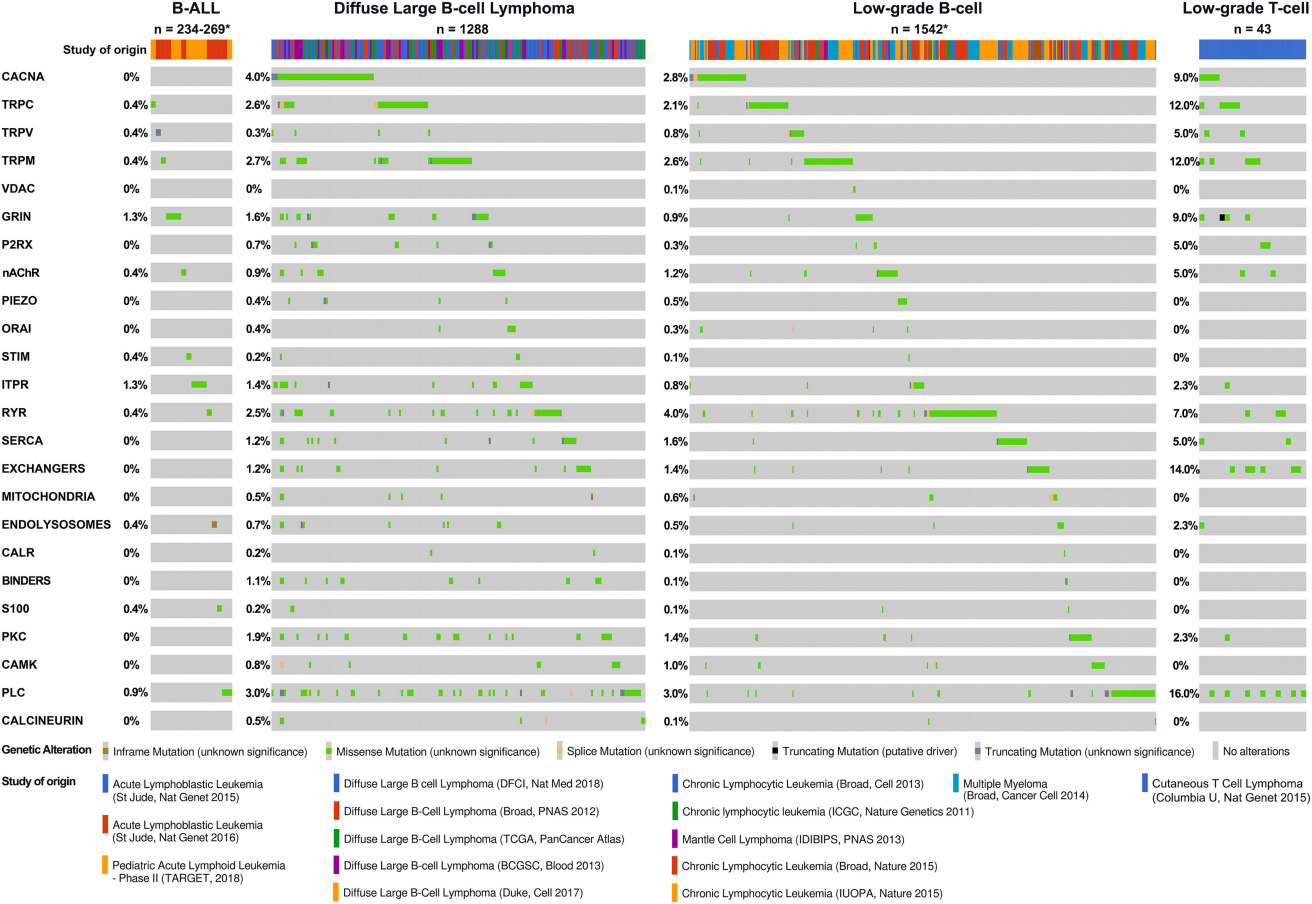
Calcium-toolkit mutations in lymphoid neoplasms. Oncoprints are shown generated using cBioportal for Cancer Genomics platform (https://www.cbioportal.org/). Events were analyzed per patient, frequencies are listed. Unaltered columns and whitespaces between columns are not shown. The Ca^2+^-toolkit genes were grouped according to function (see **Supplemental Table 1**). The molecular profiles queried included mutations but excluded copy number variations and structural variants as these were not available for most patients. The databases analyzed are indicated in the figure and referenced below: 1) For B-ALL: Acute Lymphoblastic Leukemia databases St Jude Nat Genet 2015 (327), St Jude Nat Genet 2016 (328) and Pediatric Acute Lymphoid Leukemia Phase II TARGET 2018. TARGET data was generated by the Therapeutically Applicable Research to Generate Effective Treatments initiative and is available at https://portal.gdc.cancer.gov/projects. The St Jude database also contained 8 T-ALL, 10 AML, and 5 unspecified leukemias - none had relevant mutations and these cases were excluded from the total. There were no other T-ALL cases with mutational data available for analysis so this cancer type could not be analyzed further. 2) For DLBCL: Diffuse Large B cell Lymphoma databases DFCI Nat Med 2018 (329), Duke Cell 2017 (330), Broad PNAS 2012 (331), TCGA PanCancer Atlas (332–340), and BCGSC Blood 2013 (341). 3) For low-grade B-cell neoplasms: Chronic Lymphocytic Leukemia databases Broad Cell 2013 (342), Broad Nature 2015 (343), IUOPA Nature 2015 (344), ICGC Nature Genetics 2011 (345), Mantle Cell Lymphoma database IDIBIPS PNAS 2013 (346), and Multiple Myeloma database Broad Cancer Cell 2014 (347). 4) For low-grade T-cell neoplasms: Cutaneous T Cell Lymphoma database Columbia U Nat Genet 2015 (348). Only patients with the appropriate diagnoses were selected. Specific cases analyzed are listed in **Supplemental Table 2**. The cBioPortal queries can be retrieved at the following links: B-ALL https://bit.ly/3Q9ZGvv; DLBCL https://bit.ly/3CZJpq8; low-grade B-cell neoplasms https://bit.ly/3AP87Xx; low-grade T-cell neoplasms https://bit.ly/3TvDZsT. Specific genetic variants can be found through these links, all were of unknown significance. *Numbers of patients analyzed and disease groups are clarified in **Table 3**.

**Figure 7:**
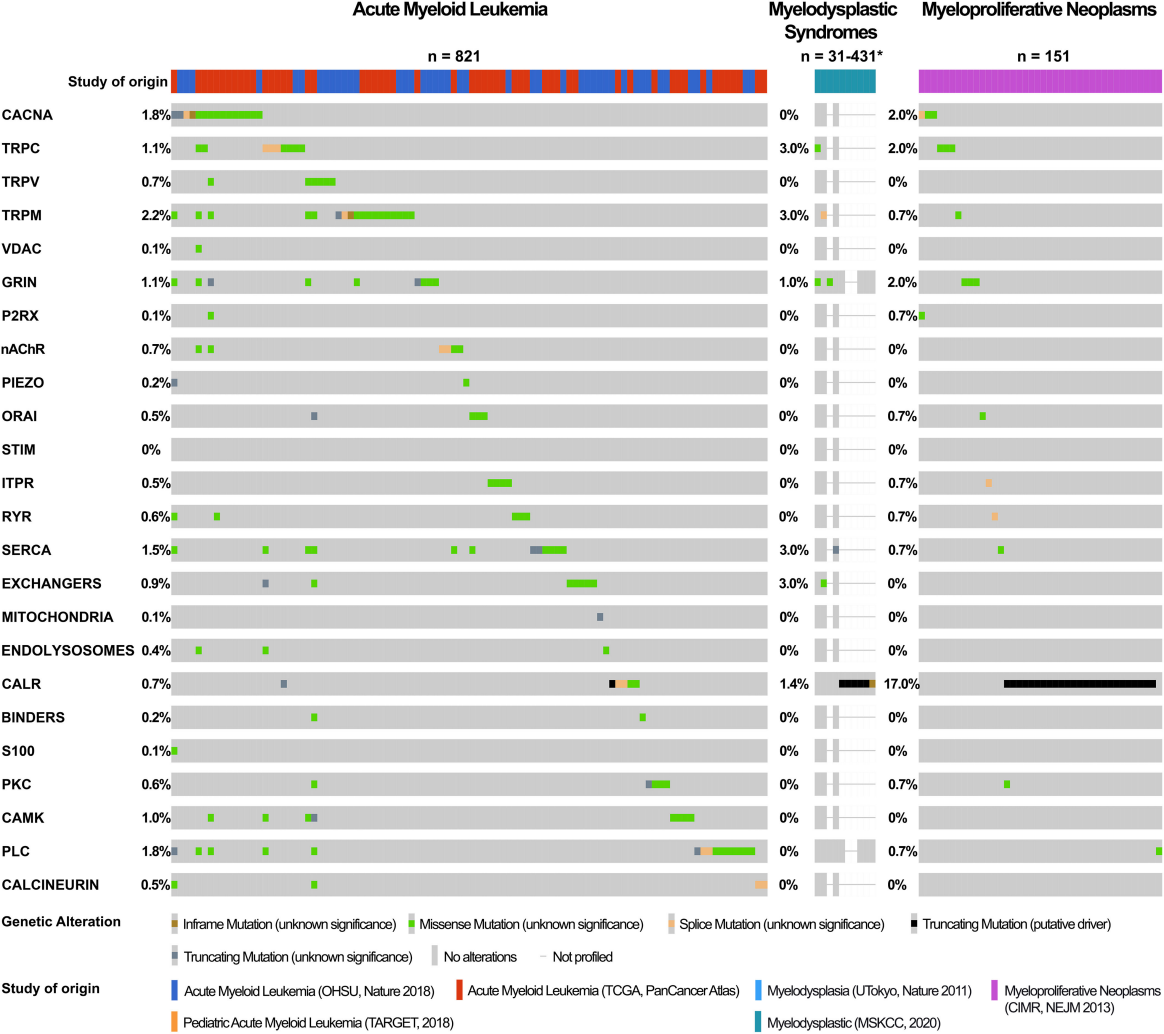
Calcium-toolkit mutations in myeloid neoplasms. Oncoprints are shown generated using cBioportal for Cancer Genomics platform (https://www.cbioportal.org/). Events were analyzed per patient, frequencies are listed. Unaltered columns and whitespaces between columns are not shown. The Ca^2+^-toolkit genes were grouped according to function (see **Supplemental Table 1**). The molecular profiles queried included mutations but excluded copy number variations and structural variants as these were not available for most patients. The databases analyzed are indicated in the figure and referenced below: 1) Acute Myeloid Leukemia databases OHSU Nature 2018 (349); TCGA PanCancer Atlas (332–340), and Pediatric Acute Myeloid Leukemia TARGET 2018. TARGET data was generated by the Therapeutically Applicable Research to Generate Effective Treatments initiative and is available at https://portal.gdc.cancer.gov/projects. 2) Myelodysplasia databases UTokyo Nature 2011 (350)), MSKCC 2020 (349, 351, 352). 466 MDS patients were excluded from the analysis as they were not profiled for any queried genes. 3) Myeloproliferative Neoplasms database CIMR NEJM 2013 (353). Only patients with the appropriate diagnoses were selected. Specific cases analyzed are listed in **Supplemental Table 3**. The cBioPortal queries can be retrieved at the following links: AML https://bit.ly/3Ts6NlX; MDS https://bit.ly/3AuvJkc; MPN https://bit.ly/3q1TZ8C. Specific genetic variants can be found through these links, apart from *CALR* mutations all were of unknown significance. *Numbers of patients analyzed are clarified in **Table 3**.

We reviewed publically available genetic cancer databases for mutations affecting molecules described in this review across the main blood cancer types. **Figure 6** demonstrates the mutational landscape in lymphoid cancers and **Figure 7** in myeloid cancers. We obtained this data using cBioPortal for Cancer Genomics platform (https://www.cbioportal.org/) (354, 355). The Ca^2+^-toolkit encoding genes were queried as gene sets grouped according to function (see **Supplementary Table S1**). Results are observational only and require validation but are useful to generate hypotheses for future research and to assist discussion. The datasets available for interrogation and the samples reviewed are listed in **Supplementary Tables S2** and **S3**. The lymphoid neoplasms reviewed included B-ALL (n = 234-269 patients depending on the gene), DLBCL (n = 1288), low-grade B-cell neoplasms (n= 1542) including CLL (n = 1254), monoclonal B-cell lymphocytosis (n = 54), mantle cell lymphoma (n = 29), plasma cell myeloma (n= 205) and low-grade T-cell neoplasms (n = 43) including Sezary syndrome (n = 26), primary cutaneous CD8/CD30 positive lymphomas (n = 14) and mycosis fungoides (n = 3) (**Figure 6**, **Table 3** and **Supplementary Table 2**). Of these lymphoid cancers, patients with B-ALL had the lowest frequency of variants in the Ca^2+^-toolkit encoding genes (5.9%) and patients with low-grade T-cell neoplasms had the highest frequency (48.8%), most carrying multiple variants (**Figure 6** and **Table 3**). The *GRIN* genes encoding NMDA receptor subunits and the *ITPR* genes encoding IP3Rs were mutated in 1.3% of B-ALL patients each, other variants were present in <1% of B-ALL patients. The cBioPortal data did not yet contain results of Bohannan et al. published earlier this year that reported the presence of *GRIN2C* mutations in high-risk B-ALL patients (145) (**Figure 3C**). It would be interesting to review these data when it becomes publically available, and pursue similar analysis in larger studies in the future.

**Table 3:** Frequencies of genetic variants in the calcium-toolkit encoding genes documented in lymphoid and myeloid cancer databases.

Cancer type	Lymphoid								Myeloid		
	B-cell							T-cell	AML	MDS	MPN
	B-ALL	DLBCL	Low-grade B-cell neoplasms					Low-grade T-cell neoplasms			
			Total	CLL	MBL	MCL	PCM				
Total number of patients per cancer type	234 except ^1^269	1288	1542	1254	54	29	205	43	821	31 except ^2^209 ^3^431 ^4^33	151
Gene sets analyzed	% of patients with gene variants in each cancer type										
CACNA	0.0	3.8	2.8	2.2	5.6	0.0	6.3	9.3	1.8	0.0	2.0
TRPC	0.4	2.6	2.1	2.3	1.9	0.0	1.5	11.6	1.1	3.2	2.0
TRPV	0.4	0.3	0.8	0.6	0.0	3.4	2.0	4.7	0.7	0.0	0.0
TRPM	0.4	2.7	2.6	1.9	3.7	10.3	5.4	11.6	2.2	3.2	0.7
VDAC	0.0	0.0	0.1	0.0	0.0	0.0	1.0	0.0	0.1	0.0	0.0
GRIN	1.3	1.6	0.9	0.8	0.0	3.4	1.5	9.3	1.1	1.0^2^	2.0
P2RX	0.0	0.7	0.3	0.2	0.0	0.0	1.0	4.7	0.1	0.0	0.7
nAChR	0.4	0.9	1.2	0.8	1.9	3.4	3.4	4.7	0.7	0.0	0.0
PIEZO	0.0	0.4	0.5	0.5	0.0	0.0	0.5	0.0	0.2	0.0	0.0
ORAI	0.0	0.4	0.3	0.1	0.0	0.0	2.0	0.0	0.5	0.0	0.7
STIM	0.4	0.2	0.1	0.0	0.0	0.0	0.5	0.0	0.0	0.0	0.0
ITPR	1.3	1.4	0.8	0.6	1.9	0.0	2.0	2.3	0.5	0.0	0.7
RYR	0.4	2.5	4.5	3.8	0.0	0.0	10.2	7.0	0.6	0.0	0.7
SERCA	0.0^1^	1.2	1.6	1.4	3.7	0.0	2.0	4.7	1.5	3.2	0.7
Calcium exchangers	0.0	1.2	1.4	1.1	1.9	0.0	2.9	14.0	0.9	3.2	0.0
Mitochondrial calcium regulators	0.0	0.5	0.6	0.8	0.0	0.0	0.0	0.0	0.1	0.0	0.0
Endolysosomal calcium channels	0.4	0.7	0.5	0.3	0.0	3.4	1.0	2.3	0.4	0.0	0.0
CALR	0.0	0.2	0.1	0.1	0.0	0.0	0.0	0.0	0.7	1.4^3^	16.6
Calcium binders	0.0^1^	1.1	0.1	0.1	0.0	0.0	0.5	0.0	0.2	0.0	0.0
S100 family	0.4	0.2	0.1	0.2	0.0	0.0	0.0	0.0	0.1	0.0	0.0
PKC	0.0	1.9	1.4	1.2	0.0	0.0	3.4	2.3	0.6	0.0^4^	0.7
CAMK	0.0	0.8	1.0	0.9	0.0	0.0	2.4	0.0	1.0	0.0	0.0
PLC	0.9	3.4	3.2	3.1	1.9	0.0	4.9	16.3	1.8	0.0^2^	0.7
Calcineurin	0.0	0.5	0.1	0.1	0.0	0.0	0.5	0.0	0.5	0.0	0.0
Total % of patients with genetic variants^5^	5.9^1^	13.9	23.0	20.3	22.2	24.1	39.0	48.8	11.9	2.3^3^	26.5

This table provides a summary of data displayed in **Figures 6**, **7**. The gene sets are listed in **Supplemental Table 1** and the full list of cases reviewed is provided in **Supplemental Table 2** (lymphoid cases) and **Supplemental Table 3** (myeloid cases). ^1,2,3,4^ Patient numbers varied for these groups from the overall total as shown. ^5^Many patients had multiple genetic variants. AML, acute myeloid leukemia; B-ALL, acute lymphoblastic leukemia; CLL, chronic lymphocytic leukemia; DLBCL, diffuse large B-cell lymphoma; MCL, mantle cell lymphoma; MBL, monoclonal B-cell lymphocytosis; MDS, myelodysplastic syndrome; MPN, myeloproliferative neoplasms; PCM, plasma cell myeloma.

In mature T-cell neoplasms, the most affected genes were those coding for PLC (16.3%), Ca^2+^/Na^+^/K^+^ exchangers (14.0%), TRPC and TRPM channels (11.6% each), Ca_V_ and NMDA receptor channels (9.3% each), ryanodine receptors (RYR) (7.0%), and a few others at 4.7% each (TRPV, P2RX, and SERCA) (**Figure 6** and **Table 3**). This particularly high frequency of variants in cutaneous T-cell neoplasms requires confirmation in larger cohorts. Unfortunately, no other mature or precursor T-cell malignancies could be reliably reviewed. Less than 10 T-ALL samples with mutational data were identified and none had relevant mutations. Ca^2+^ signaling is critical for T-cell activation downstream of TCR and linked closely with the regulation of T-cell metabolism (356). Multiple studies highlighted the role of Orai, Ca_V_, TRP, NMDA receptors and other Ca^2+^ regulators in normal and malignant T-cells (134, 192, 357–361). Further studies are required to examine potential contribution of these changes in T-cell cancers.

In mature B-cell neoplasms, mutated genes were broadly similar between the low-grade and high-grade cancers (**Figure 6** and **Table 3**). The frequency of variants was higher in patients with low-grade B-cell neoplasms, 23.0% compared with 13.9% in DLBCL, but many patients with DLBCL had multiple variants. The types of variants were similar between patients with CLL, monoclonal B-cell lymphocytosis, mantle cell lymphoma and plasma cell myeloma. In low-grade B-cell lymphoproliferative disorders, variants in *RYR1-3* were the most common (4.5%), followed by *PLC* (3.2%), *CACNA* (2.8%), *TRPM* (2.6%) and *TRPC* genes (2.1%), with others present in <2% of patients. Some of these mutations appeared exclusive between each other. In DLBCL, *RYR* mutations were less common (2.5%) but *CACNA* variants were more common (3.8%) (**Figure 6** and **Table 3**). One could hypothesize that the acquisition of multiple mutations associates with a higher grade. Further bioinformatics analysis and laboratory studies to determine the role of gene variants in lymphoid cancers appears warranted. For example, experimental studies to examine the contribution of mutations in plasma membrane Ca^2+^ channels and RYRs would be of interest. The existing literature in this area is limited. RYRs facilitate the release of Ca^2+^ from the ER in addition to IP3Rs (362) (**Figure 1**). They are the largest ion channels known regulated by Ca_V_1.1/Ca_V_1.2-mediated Ca^2+^ entry, as well as other small molecules including ATP, calmodulin, calsequestrin and CaMKII (363). RYR1 is primarily expressed in skeletal muscles but is also present in B-lymphocytes (364, 365) and Burkitt lymphoma-derived Namalwa cells (366). The RYR role in B-cell function remains unclear but its activity downregulates CD38 expression (366). High levels of CD38 associate with poor risk CLL (367). Studies to determine the mechanism of the RYR contribution to the regulation of CD38 and the impact of RYR mutations on CD38 expression and B-cell activation may be helpful.

In the group of myeloid neoplasms, we reviewed 821 patients with AML, 151 with MPN and 31-431 patients with myelodysplastic syndrome (MDS) (depending on the gene) (**Figure 7**, **Table 3** and **Supplementary Table S3**). Compared with B-ALL, AML patients carried more variants (11.9% versus 5.5% in B-ALL patients). Variants in *CACNA* (1.8%) and *TRP* genes (2.2% in *TRPM*, 1.1% in *TRPC*, 0.7% in *TRPV*) were the most common in AML but no variants exceeded 3% frequency. MDS patients had a lower frequency of variants at 2.3% overall, although data for these patients was limited. In the MPN group, 17% had *CALR* mutations, consistent with it being a known MPN driver (353, 368). In addition, a further 9.5% of MPN patients had variants in other Ca^2+^-toolkit genes (including 2.0% in *CACNA*, *TRPC* and *GRIN* genes each). It was intriguing that these variants were exclusive with *CALR* mutations and also appeared exclusive with each other, raising the possibility of their independent effects in MPN (**Figure 7** and **Table 3**). Evidence slowly accumulates that Ca^2+^ signaling is aberrant in *CALR*-mutated MPN (9, 290, 291, 319). The pattern of the mutational landscape revealed by our review argues that further work into Ca^2+^ signaling in MPN is warranted, including in patients without *CALR* mutations.

## 6 Concluding remarks

In all cell types, including hematopoietic, Ca^2+^ is an essential second messenger controlling a wide range of cellular functions, including activation of gene transcription, protein kinase signaling, cell cycle, cell survival, proliferation, differentiation, migration, and apoptosis (2–4). Others have reviewed deregulation of Ca^2+^ signaling in specific cancer subtypes such as AML, CLL and plasma cell myeloma, and summarized the influence of Ca^2+^ remodeling on cell proliferation and differentiation (369–371). Our review is the first one, to our knowledge, that highlights the extent and intricacies of alterations in the Ca^2+^-toolkit in a wide range of hematologic cancers. We also provide a review of blood cancer databases for genetic variants in the Ca^2+^-toolkit encoding genes.

**Figure 8:**
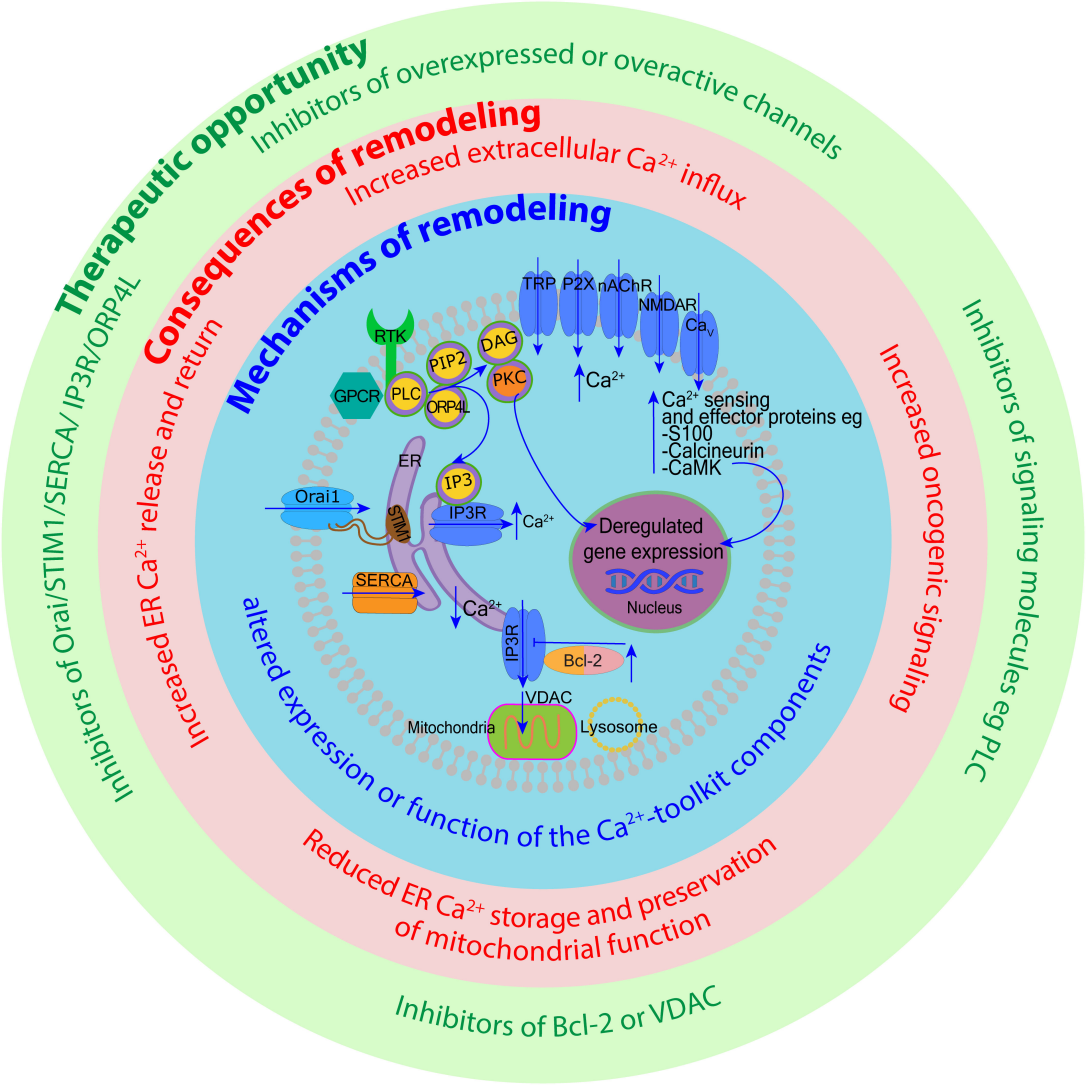
Remodeling of calcium signaling in hematologic cancers and therapeutic opportunities this presents. Examples of mechanisms responsible for the remodeling of Ca^2+^ signaling in blood cancer are depicted within the central blue circle. Their direct consequences are listed in the middle red circle, and therapeutic opportunities arising from these changes are highlighted in the outer green circle. For example, the increased expression of the plasma membrane Ca^2+^ channels (a mechanism of remodeling) leads to increased extracellular Ca^2+^ influx supporting oncogenic signaling (a consequence). Such changes could be counteracted by specific Ca^2+^ channel inhibitors (a therapeutic opportunity). In another example, mechanisms that spare cancer cells from mitochondrial Ca^2+^ overload (e.g. through the overexpression of Bcl-2 or VDAC) could be counteracted by inhibitors of these molecules. The design of novel therapies heavily relies on our understanding of the Ca^2+^-toolkit remodeling in different blood cancers.

It has been well documented that in solid tumors, cancer cells remodel Ca^2+^ signaling to enhance cancer hallmarks (1, 2). Our review emphasizes that similar alterations occur in blood cancer, driven by changes in expression, function and possibly mutations in the Ca^2+^-toolkit components. **Figure 8** provides a schematic summary of the underlying mechanisms and their consequences in blood cancer cells. Normal cells in response to activation mostly use Ca^2+^ released from the ER to support signaling. However, prolonged IP3R-mediated Ca^2+^ release in chronically activated cancer cells (e.g. due to oncogenic mutations) may lead to mitochondrial Ca^2+^ overload triggering apoptosis. Therefore, to escape apoptosis, cancer cells shift to preferentially utilize extracellular Ca^2+^ transferred into cells by overexpressed or overactive plasma membrane Ca^2+^ channels. On the other hand, mechanisms that utilize ER-derived Ca^2+^ may be reduced, and overactive SERCA2 pumps Ca^2+^ back into the ER. This remodeling of Ca^2+^ signaling helps cells maintain heightened oncogenic signaling while shielding mitochondria from Ca^2+^ overload. The examples of mechanisms providing anti-apoptotic effects include Bcl-2-mediated inhibition of IP3R in DLBCL and reduced ER Ca^2+^ binding by mutated CALR in MPN. In addition, intracellular mediators of Ca^2+^ signaling may be overexpressed or overactive e.g. PLC, PKC, ORP4L, calcineurin and CaMK. Understanding how Ca^2+^ signaling remodels in cancer cells creates therapeutic opportunities, with the potential to spare normal cells and tailor therapies according to the underlying mechanism in different cancers/patients (**Figure 8**).

Research into Ca^2+^ signaling in blood cancer has become very active in recent years. Many critical discoveries have been made but multiple challenges remain. We highlighted some areas for future investigation throughout this review, including the need to characterize diverse mechanisms of Ca^2+^ remodeling and determine the significance of the mutational landscape affecting the Ca^2+^-toolkit genes in different cancer types. Such work is not easy. Ca^2+^ signaling is a complex network of intertwining pathways and ubiquitous for cellular functioning. It can be difficult to identify the causes and consequences of the changes found in blood cancer. Most of the previous studies focused on a specific mechanism of Ca^2+^ signaling in isolation and used cell lines to characterize it. Moving forward, we should consider changes to the entire Ca^2+^ toolkit and use multiple disease models *ex vivo* and *in vivo* to study effects of multiple gene networks in cancer cells and stromal cells. This will require innovative approaches and collaboration between experts in Ca^2+^ signaling, hematological sciences and clinical hematologists.

The ultimate aim of pursuing research in this area is to improve the treatment of patients. Calcium pathways are amenable to modulation and may offer novel points for therapeutic intervention. The main targets/pathways were recently summarized for AML (371). Our **Table 4** provides a range of examples of compounds/drugs that have been found to exert an impact on the functional outcome *in vitro* or *in vivo* in diverse blood cancer types. Some of these compounds are in clinical use for other applications or undergo pre-clinical/clinical testing in solid tumors (394, 395). If their targets are found to be pathogenic in blood cancer, these drugs could be rapidly transitioned to hematologic applications.

**Table 4:** Demonstrative examples of calcium-related compounds/drugs (specific inhibitors or activators of calcium channels and receptors) that have been found to exert an impact on the functional outcome *in vivo* or *in vitro* in diverse blood cancer types.

Target	Compound	Functional effects	Cancer type	References
**Plasma membrane Ca^2+^ channel activators**				
TRPV2	Cannabidiol	↑ cytoplasmic [Ca^2+^], ↑ ROS production, induces mitophagy, ↓ cell viability, ↓ cell proliferation, ↓ cell cycle progression	CML ^(C)^	(147)
			Plasma cell myeloma ^(C)^	(372)
NMDAR	Glutamate	↑ cytoplasmic [Ca^2+^], ↑ cell proliferation	AML ^(C)^	(182, 183, 373)
	NMDA	↑ cytoplasmic [Ca^2+^], ↑ cell proliferation	AML ^(C)^	(182, 183, 373)
**Plasma membrane Ca^2+^ channel inhibitors**				
P2X7	A740003	↓ cell proliferation, ↓ self-renewal of leukemia-initiating cells, ↑ survival time	AML ^(C,M)^	(124, 125)
	AZ10606120	↓ cytoplasmic [Ca^2+^] influx, ↓ leukemic growth	AML ^(P,M)^	(126)
TRPC3	Pyr3	Pyr3 and Dex - co-treatment: ↓ Dex-mediated Ca^2+^signaling, ↑ cell death, ↑ cell cycle arrest, ↑ apoptosis, ↑ mitochondrial membrane potential depolarization, ↑ ROS production	ALL ^(P,C)^	(374)
TRPV2	Tranilast	↓ cytoplasmic [Ca^2+^], ↓ cell growth, ↑ apoptosis, ↑ cell cycle arrest	AML ^(C)^ CML ^(C)^ Non-Hodgkin lymphoma ^(C)^	(127)
α7-nAChR	Methyllycaconitine citrate	↓ cytoplasmic [Ca^2+^], ↓ proliferation	CML ^(C)^	(177)
	NBP-14	↓ migration, ↓ α7-nAChR expression	AML^(C)^ CLL ^(C,P)^ Plasma cell myeloma ^(C)^	(151)
NMDA receptor	Memantine	↓ cytoplasmic [Ca^2+^], ↓ proliferation, ↓ cell viability, facilitates differentiation, inhibits proplatelet formation, alteres expression of Ca^2+^ channels and pumps, ↑ cytarabine-mediated cell killing	AML ^(C)^	(182, 183, 373)
	MK-801	↓ cytoplasmic [Ca^2+^], ↓ cell proliferation, ↓ cell viability, facilitates differentiation, inhibits proplatelet formation	AML ^(C)^	(182, 373)
VDAC	VDAC-based (decoy) peptides	mitochondrial dysfunction, ↓ ATP production, mitochondrial Ca^2+^ overload, cytochrome c release and apoptosis	ALL ^(C)^	(188, 192)
	Avicin	as above	ALL ^(C)^	(192, 375)
Ca_V_1.2	Lercanidipine	↓ Ca ^2+^ influx into AML-MSCs, ↓ proliferation of AML-MSCs and of AML blasts, sensitizes leukemia cells to other drugs	AML ^(P,M)^	(138)
Ca_V_3	mibefradil and NNC-55-0396	↓ cytoplasmic [Ca^2+^], ↓ proliferation, ↑ apoptosis	ALL	(376)
**ER/SOCE Ca^2+^ channel and effector modulators**				
Broad inhibitor of SOCE*	BTP-2	↓ megakaryocyte proliferation	CALR-mutated MPN	(9)
Bcl2 inhibitor	BIRD-2	Disrupts the Bcl2-IP3R interaction, ↑cytoplasmic [Ca^2+^] through IP3R, ↑ apoptosis	CLL ^(C,P)^	(154, 226–228)
			DLBCL ^(C)^	(153–155, 225, 228)
			Follicular lymphoma ^(C)^	(227)
			Plasma cell myeloma ^(C)^	(227)
IP3R	Wogonoside – IP3R1 activator	↑ cytoplasmic [Ca^2+^], increases differentiation, induces cell cycle arrest, ↓ cell viability, ↓ STAT3 activation	AML ^(C,M,P)^	(377, 378)
			ALL ^(C,M)^	(379)
	Xestospongin – IP3R inhibitor	Inhibits Ca^2+^ release into cytoplasm, ↑ cell death, synergy with Dex to further ↑cell death	ALL ^(C)^	(380)
Orai1 inhibitor	RP4010	↓ cell proliferation	AML ^(C,P,M)^	(381)
Orai1 inhibitor	Synta66	↓ cell proliferation	CLL ^(C,P)^	(11)
Orai3 activator	Tipifarnib	↑ cytoplasmic [Ca^2+^], loss of membrane integrity, ↑ cell death	AML ^(C)^	(382)
			Plasma cell myeloma ^(C)^	(382)
ORP4L inhibitor	LYZ-81	↓ Ca^2+^ oscillations, ↑ cell death of LSCs, ↓ PIP2 hydrolysis	AML ^(C,P,M)^	(211)
		↑ cell death, ↓leukemic engraftment, ↑ survival	Adult T-cell leukemia ^(P,M)^	(383)
PLC inhibitor	U73122	↓ cytoplasmic [Ca^2+^], ↑ cell death,	CLL ^(P)^ DLBCL ^(C)^	(154)
CaMKII inhibitor	berbamine	Eliminates CML LSCs	CML ^(P,M)^	(310)
SERCA inhibitor	CAD204520	↑ cytoplasmic [Ca^2+^], ↓ cell viability, ↑ cell cycle arrest, targets mutated NOTCH1, SERCA inhibition achieved *in vivo* without cardiac toxicity	Mantle cell lymphoma ^(C)^	(384)
			ALL ^(C,P,M)^	(384)
	Casearin J	↑ cytoplasmic [Ca^2+^] *via* ER and SOCE activation, ↑ ROS production, ↓ cell viability, inhibits NOTCH1 signaling	ALL ^(C)^	(385, 386)
	CXL017	↓ cell viability	AML ^(C)^	(387)
	HA14-1 (also binds hydrophobic cleft of Bcl-2)	↑ cytoplasmic [Ca^2+^], has adverse effects on platelet survival (388)	ALL ^(C)^	(389, 390)
			CLL ^(P)^	(391)
			DLBCL ^(C)^	(391)
	JQ-FT	↓ cell viability, ↑cell cycle arrest, ↓ proliferation, targets mutated NOTCH1	ALL ^(C,M)^	(392)
	Thapsigargin	↑ cytoplasmic [Ca^2+^], ↓ cell viability, ↓ cell size, ↑ cell cycle arrest, targets mutated NOTCH1 cell lines, impairs cardiac cell mechanics	ALL ^(C,M)^	(253, 384–386)
**Endo-lysosomal Ca^2+^ modulators**				
TPC1/2 inhibitor	Tetrandrine	↓ cell proliferation, ↑ cell death	AML ^(C,P)^	(233–236)
		Inhibits cytotoxic drug sequestration in the lysosomes which helps overcome chemoresistance	ALL ^(C,P)^	(237)
Co-localizes with lysosomes	Imipramine blue + pimozide (STAT5 inhibitor)	↑ cytoplasmic [Ca^2+^], loss of mitochondrial membrane potential, liberation of ROS, ↑ apoptosis	AML ^(C,P)^	(240, 393)

References were prioritized that include data on the effects on calcium signaling. ↑ = increased, ↓ = decreased. *Broad inhibitors of SOCE were used in many studies. Their effects are not listed but this particular example has been included to reflect the emerging therapeutic potential for the modulation of SOCE in CALR-mutated MPN. (P) = Patient cells, (C) = Cell lines, (M) = Mouse model. ALL, acute lymphoblastic leukemia; AML, acute myeloid leukemia; CLL, chronic lymphocytic leukemia; CML, chronic myeloid leukemia; Dex, dexamethasone; DLBCL, diffuse large B-cell lymphoma; LSCs, leukemia stem cells. MPN, myeloproliferative neoplasms.

In conclusion, multiple Ca^2+^ homeostatic mechanisms and Ca^2+^ responsive pathways are altered in hematologic cancers. Some of these alterations may have genetic basis, including in MPN, B-cell and T-cell lymphoproliferative disorders, but studies are limited. Most changes in the Ca^2+^-toolkit do not appear to define or associate with specific cancer types but may influence variables such as grade (e.g. in mature B-cell neoplasms), prognosis including responsiveness to chemotherapy (e.g. in ALL, AML and CLL), and complications (e.g. thrombosis and bone marow fibrosis in MPN). Deregulation of Ca^2+^signaling provides an opportunity to design novel therapeutic interventions. Some options are currently investigated mostly at the pre-clinical level in various cancer models (e.g. of AML, ALL and DLBCL). Similar opportunities are being considered in solid tumours, which may facilitate faster clinical translation to blood cancer. Future research to define the role of specific Ca^2+^ regulatory mechanisms in different blood cancer types will be challenging but such work is likely to advance therapies.

## Author contributions

MLK-Z conceived and designed the work. TI and MLK-Z wrote most of the paper. JL and TNG helped with the literature review, figure preparation, and writing of specific sections; JL wrote the TRP section, TNG wrote about VDACs and helped analyze the cBioPortal data. AB provided advice and wrote about calcium homeostasis in mature red cells. All authors contributed to the article and approved the submitted version.

## Funding

Bone Marrow Cancer Research Trust (Christchurch) (UoA 9102-3720536) and Auckland Medical Research Foundation (Funder reference 1119009) provided salary funding to staff working on this project but had no influence over any aspects of the work or the decision to publish.

## Conflict of interest

The authors declare that the research was conducted in the absence of any commercial or financial relationships that could be construed as a potential conflict of interest.

## Publisher’s note

All claims expressed in this article are solely those of the authors and do not necessarily represent those of their affiliated organizations, or those of the publisher, the editors and the reviewers. Any product that may be evaluated in this article, or claim that may be made by its manufacturer, is not guaranteed or endorsed by the publisher.
